# Dynamic transcriptional and epigenetic changes define postnatal tendon growth

**DOI:** 10.1371/journal.pgen.1011902

**Published:** 2025-11-18

**Authors:** Heather L. Dingwall, Mor Grinstein, Benjamin Peterson, Terence D. Capellini, Jenna L. Galloway

**Affiliations:** 1 Department of Human Evolutionary Biology, Harvard University, Cambridge, Massachusetts, United States of America; 2 Department of Orthopedic Surgery, Massachusetts General Hospital, Harvard Medical School, Boston, Massachusetts, United States of America; 3 Harvard Stem Cell Institute. Bauer Building, Cambridge, Massachusetts, United States of America; 4 Renal Division, Brigham and Women’s Hospital, Harvard Medical School, Boston, Massachusetts, United States of America; 5 The Broad Institute of Harvard and MIT, Cambridge, Massachusetts, United States of America; University of Michigan, UNITED STATES OF AMERICA

## Abstract

Tendons are dynamic structures that efficiently transmit force and enable movement. From birth, tendons undergo dramatic changes from a principally cellular tissue to a hypocellular one characterized by a dense and highly ordered extracellular matrix. During this time, tendon cells change morphology from rounded to stellate in appearance and their proliferative rates decline. Significant expansion and maturation of the extracellular matrix (ECM) grow the tendons in length and diameter and alter their biomechanical properties to sustain increased physical activities. Surprisingly, for such an important stage of tendon maturation, we understand very little about the transcriptional and epigenetic regulators that direct these processes. Here, we present a roadmap of genes that are differentially regulated during the early neonatal and postnatal time period. We find differentially expressed genes fall into specific transcriptional modules, representing expression increases, decreases, or gene sets undergoing dynamic changes over postnatal time. By pairing our transcriptomic data with epigenetic data, we performed an integrative analysis of the datasets and further defined modules with highly correlated changes in gene expression and chromatin accessibility. From this analysis, several new pathways emerge. Among them, we focus on Yap1, a transcriptional co-activator of the Hippo signaling pathway. We observe accessible regions near to differentially expressed genes, containing motifs for TEAD, the transcription factor that binds Yap to regulate transcription. Conditional loss of *Yap1* at postnatal stages alters early expression of *Col1a1* and matrix organization and density but does not affect gross ultrastructural and mechanical properties at later stages. Together, our analyses identify a regulator of early matrix formation and provides a rich dataset with which to interrogate transcriptional networks and pathways during this poorly understood time in tendon growth.

## Author summary

Tendons provide essential connections within our musculoskeletal system, transmitting the force from muscle to bone to enable movement. Concomitant with skeletal growth, tendon development continues through postnatal stages and is characterized by several major changes in the structure and organization of the tendon extracellular matrix (ECM), regenerative capacity, and mechanical strength. Remarkably, we understand very little about the molecular changes that characterize this important period of tendon growth and maturation. By examining global changes in gene expression and genome organization, we identify distinct modules of gene and chromatin regulation that change dynamically over this developmental timeframe. We specifically identify differentially regulated regions that may be controlled by components of the Hippo signaling pathway, which are nearby tendon ECM genes. We also find that loss of a transcriptional co-regulator of Hippo signaling affects early stages of tendon ECM gene expression but does not impact later tendon ECM maturation. Altogether, this work provides a rich resource with which to understand this key developmental period of tendon growth and maturation.

## Introduction

Tendons are crucial components of the musculoskeletal system that are composed of a highly ordered extracellular matrix (ECM), predominantly made of type I collagen fibrils organized in a multiscale hierarchical structure. This structured ECM is critical to their ability to efficiently transfer force from muscle to bone. Adult tendons are prone to injury, from acute rupture to chronic degeneration. Following injury, tendons undergo a slow, scar-mediated healing process, resulting in imperfect tissue repair [1]. Despite advances in surgical intervention for tendon injuries, the repaired tissue remains mechanically compromised due to this form of healing, yielding a high failure rate after surgery [2,3]. A greater understanding of tendon formation and growth would be instrumental towards developing regenerative based therapies for treating their injury and disease.

Several transcription factors (TF) and signaling pathways are vital to proper tendon formation during embryogenesis. Among them, Scleraxis (Scx) is a basic helix-loop-helix TF that is the earliest marker of tendon cell fate and is required for formation of force-transmitting limb tendons [4,5]. Mohawk (Mkx) and the Early Growth Response-like (Egr) TFs Egr1 and Egr2 are expressed at slightly later embryonic stages and are required for tendon matrix maturation [6,7]. All four of these TFs regulate the production of major collagens (Col1a1, Col1a2, Col3a1) and tenomodulin (Tnmd), a transmembrane glycoprotein found on differentiating tendon cells [6–11]. TGF-β signaling also plays an important role in embryonic tendon formation through the regulation of collagens, other ECM proteins [12–14], and maintaining *Scx* expression [14–18].

Postnatal tendon growth is characterized by structural and compositional changes to the extracellular matrix (ECM) that result in a highly organized, matrix-rich mature tissue [19–21]. Both linear and lateral growth in the tendon are primarily driven by rapid expansion of the ECM rather than by cell proliferation [20–22]. Although there is detectable cell proliferation in early postnatal days (P) 0–14, proliferation decreases significantly by P21 and is maintained at an extremely low rate from 1 month to 1–2 years of life [23]. Interestingly, this transition in cell proliferation and matrix maturation correlates with the loss in regenerative potential in mammalian tendons. For example, 1–2 week-old mice exhibit regenerative-like healing with considerably improved mechanical properties when compared with 3-week-old [24] and 4–5-month-old mice [25]. Tendon healing occurs without the formation of a lasting fibrotic scar in both fetal [2,26] and early postnatal stages [19,25]. This regenerative ability is retained even when the immature tendon is wounded after transplantation into the adult environment [2,26], suggesting the regenerative potential is intrinsic to the tendon. However, this regenerative ability is restricted to fetal and early postnatal stages [2,25]. A similar decline in regenerative ability has been observed in other organs, such as the heart. After injury, cardiac muscle regenerates prior to 7 days of neonatal development, but no regeneration is observed at later stages [27]. Notably, this transition coincides with the developmental stage at which the cardiomyocytes lose their ability to proliferate [28]. Interestingly, tendon cells also decrease in proliferative activity after 7–14 days postnatally, correlating with the transition from regenerative to reparative healing and increase in mechanical properties [24,25]. Thus, there appears to be similar timing in the loss of cell cycle activity and of regenerative potential that correlates with the stages at which the tendon ECM significantly expands and matures mechanically.

Although several studies have described postnatal changes to the tendon ECM [19,21,23,29], fewer works have examined the molecular changes taking place in postnatal tendon cells [18,30]. The identification of gene regulatory programs that are specifically controlled during this process would be of great significance towards understanding the mechanisms by which tendon cells regulate tendon tissue growth and maturation. Some transcriptomic studies have focused on the active transcriptional programs in embryonic tendon cells [31,32] whereas others have examined gene expression differences in isolated postnatal tendon progenitor cells after expansion under *in vitro* culture conditions [33]. In addition, adult studies have examined differences in mutant mouse tendons [34] and genes involved in aging [35]. More recently, single cell RNA-sequencing (scRNA-seq) has examined tendon cell populations at postnatal stages to show that loss of TGF-β signaling leads to loss of tendon cell fate [18]. Single nucleus (sn)RNA-sequencing and Assay for Transposase Accessible Chromatin and sequencing (snATAC-seq) also identified candidate transcriptional regulators of specific tendon cell sub-populations, including potential tendon progenitor cells [30]. In addition, scRNA-seq analysis has revealed tendon cell heterogeneity at multiple stages [18,36–38]. These methods are powerful to identify distinct tendon cell populations and molecularly characterize their regulation, yet such methods capture a small portion of the tendon cell transcriptome and have lower sensitivity.

In this study, we use an integrative genomics approach to characterize tendon cells as they transition from highly proliferative to relatively quiescent stages. Specifically, we employ RNA-seq and ATAC-seq to identify expressed genes and open chromatin regions, respectively, in mouse tendon cells as they mature from neonatal stages (P0) to early adulthood (P35). Changes in chromatin accessibility throughout tendon growth can help identify temporally specific putative *cis*-regulatory elements that coordinate the transcriptome dynamics involved in postnatal tendon maturation. By integrating these transcriptomic and epigenomic signatures, we identify key genes and signaling pathways that may mediate the observed shift in tendon cell proliferation, regenerative potential, and matrix maturation. As an example of our ability to discover functionally relevant pathways, we identified the Hippo signaling pathway to be differentially regulated during P0-P35 and tested its role in postnatal tendon development. *Yap1*, a transcriptional co-activator in the Hippo signaling pathway, was specifically deleted at postnatal stages using *Scx-CreERt2*. Loss of *Yap1* in *Scx*-expressing cells affected Collagen expression, organization, and cell number at postnatal day 14, but did not impact the mechanical properties or ECM structure at later stages. Together, this research provides a rich resource of new candidate pathways to investigate in the context of tendon growth, maturation, and injury repair.

## Results

### Transcriptome dynamics during postnatal tendon growth and development

**Fig 1:**
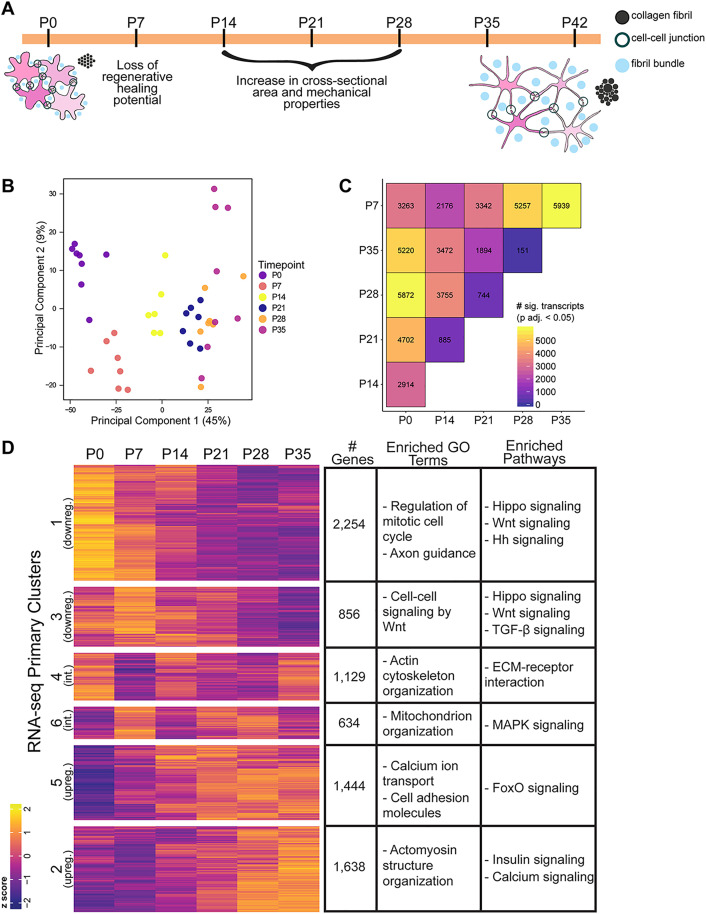
RNA-seq identifies modules of differentially co-expressed genes during postnatal tendon growth. A) Schematic representation and summary of key transitions in tendon biology during the early postnatal growth period. B) PCA of normalized counts from bulk RNA-seq shows separation of timepoints along PC1. Earlier timepoints (P0, P7, and P14) are more distinctly clustered than later timepoints (P21, P28, P35). C) Results of Wald test between all pairs of timepoints (p adj. < 0.05) show more DE genes between pairs of early timepoints compared to pairs of later timepoints. D) Clustered heatmap of genes determined to be DE at any point during the time series (P0 to P35) by likelihood ratio test (p adj. < 0.05); gene clusters were determined using the PAM algorithm. Z-scores were calculated from log2(x + 1)-transformed, quantile normalized counts per million reads (CPM). The adjacent table indicates the number of genes occupying each cluster and a selection of the top enrichments from GO and pathway analyses (p adj. < 0.05).

To focus on the molecular changes that occur during the transition in postnatal tendon growth and regenerative potential (Fig 1A), we performed RNA-seq on mRNA isolated from whole distal limb tendons, collected weekly from postnatal day (P) 0 to P35. A Principal Components Analysis (PCA) on normalized gene counts shows separation of P0, P7, and P14 samples along PC1, while samples from mice at P21 and older do not separate clearly from one another (Fig 1B). It is also notable that P35 has higher within-group variability than the other time points, which could at least partially explain the inability of this time point to separate from P28. Importantly, PCA shows that sequencing pool does not significantly influence sample clustering (S1A Fig) indicating that the group in which a sample was sequenced (sequencing pool) accounts for negligible, if any, sample variance. We also did not detect any clear separation according to sex at these stages (S1B Fig). A likelihood ratio test on gene counts using the DESeq2 framework [39] found that approximately 22% of detected genes were differentially expressed (DE) between at least two time points in the 5-week time series (p adj. < 0.05). This approach, in which two user-defined models of gene expression are assessed for their goodness of fit, allowed us to test the effects of the time component of the model on gene expression independent of RIN (see Methods), as well as investigate differences in expression among all time points simultaneously. We first investigated the specific pairwise differences in transcriptome-wide gene expression from P0 to P35 and found that many genes are differentially expressed between these three time points (Figs 1C and S1C). Given assumptions in the tendon literature that mature tendon cells exhibit relatively low metabolic activity [40], we were quite surprised by the large number of genes that were up-regulated from P0 to P35. Comparing the number of differentially expressed genes between each pair of time points shows few genes that are differentially expressed between sequential time points at older stages (i.e., after P14) compared to younger stages (Fig 1C). This suggests that the first two postnatal weeks are a period of rapid, dynamic transcriptomic change, which slows after P21, mirroring known tendon cell proliferation dynamics [23].

### Expression of known genes involved in tendon development

Next, we defined a set of “tendon” and ECM genes to investigate their expression within this data set. Because so few genes are known to be directly involved in tendon development, this set is small enough to examine the expression of each gene individually (S2 Fig). *Scx*, *Mkx*, *Egr1*, and *Egr2* are all expressed postnatally, but none are significantly DE across the stages analyzed (S2A Fig; p adj. > 0.05). *Scx* expression is highly variable among the individuals within a sample; each time point contains distinct high- and low-expressers of *Scx* (S2A Fig). While we have observed higher than expected within-sample variance in *Scx* expression before [23], the cause remains unclear.

We also investigated the expression of TGF-$\begin{array}{c} \beta \end{array}$ ligands and receptors. The TGF-$\begin{array}{c} \beta \end{array}$ pathway, and TGFβ2/3 or TGFβR2 specifically, are required for normal tendon development [14]. TGF-β signaling is also necessary for the contribution of *Scx*-lineage cells to neonatal regeneration [41]. We found that *Tgfb1* is differentially upregulated at P7 compared to the other stages (p adj. < 0.05), while *Tgfb2* and *Tgfb3* are not DE at any point (S2B Fig). TGF-$\begin{array}{c} \beta \end{array}$ receptor expression varies: *Tgfbr1* is intermittently DE, *Tgfbr2* is not DE, and *Tgfbr3* is differentially upregulated from P0 to P35 (p adj. < 0.05; S2B Fig).

All ECM related genes in this gene set were found to be significantly DE at some stage during the time series (S2C Fig; p adj. < 0.05). *Decorin* (*Dcn*) and *Fibromodulin* (*Fmod*) expression increases monotonically from P0 to P35; *Col2a1*, *Col14a1*, *Col3a1*, and *Tenomodulin* (*Tnmd*) expression decreases steadily from P0 to P35; and *Col1a1* and *Col1a2* expression peaks around P14 after which they are downregulated. Expression of the matrix gene *Biglycan* (*Bgn*) appears to be partitioned into two phases: it is more highly expressed from P0-P14 followed by downregulation by P21 (p adj. < 0.01), after which expression remains low through P35 (S2C Fig).

### Clustering analysis and expression module identification

To gain a more detailed understanding of the temporal gene expression changes that occur between P0 and P35, we performed an unsupervised cluster analysis using the partitioning around medoids (PAM) algorithm on normalized gene counts. This allowed us to discover six differential expression modules – cohorts of genes that tended to be co-expressed over the course of the six time points in the study (Fig 1D). These co-expression modules reveal 3 broad patterns of differential expression within our data: downregulation over time (Clusters 1 and 3); upregulation over time (Clusters 2 and 5); and intermittent expression (Clusters 4 and 6). The key difference between each pair of expression modules that fit a pattern is the exact timing of the expression change(s). For example, many of the genes grouped into cluster 5 are more strongly upregulated about one week earlier (~P21) than those in cluster 2 (~P28). Gene ontology (GO) enrichment analyses on each expression module suggest that biological processes involved in cell proliferation and differentiation dominate the earlier time points, while cell communication and cytoskeleton organization become more important later during postnatal development (Fig 1D).

Cluster 1, which captures downregulated genes over time, is highly enriched for GO terms related to mitosis, cell cycle, and DNA replication (p. adj < 2 x 10^-18^, q < 5 x 10^-16^). Additionally, a pathway analysis on Cluster 1 found an enrichment of genes involved in the Wnt and Hippo signaling pathways, both of which are known to be involved in regulating cell proliferation in multiple tissues. Cluster 3 contains genes that also become downregulated throughout the five-week time series with peak expression occurring around P7. Like Cluster 1, this module is enriched for genes involved in Wnt signaling, as well as Smoothened (Smo) signaling, a key component of the Hedgehog (Hh) signaling pathway. Cluster 3 is also enriched for muscle cell proliferation and differentiation.

Both modules (Clusters 2 and 5) are characterized by a pattern of steadily increasing expression from P0 to P35 and show enrichment for biological processes involved in calcium ion transport, regulation, and signaling. Clusters 2 and 5 are also enriched for muscle-related GO terms, although they differ in their specifics. Cluster 2 genes, which are upregulated at ~P28, are enriched for muscle developmental and differentiation processes, as well as actomyosin structure organization. Meanwhile, Cluster 5, which contains genes that are upregulated earlier, is enriched for GO terms related to muscle contraction and structure, in addition to processes involved in calcium ion transport (*S100a1*).

The genes belonging to Cluster 4 exhibit intermittent expression, first upregulated at P0, then moderately at P14, and later at P35. Interestingly, enrichment analyses found that the genes comprising Cluster 4 are highly enriched for processes related to the regulation of the actin cytoskeleton, ECM organization, and collagen biosynthesis, as well as small GTPase-mediated signal transduction. Multiple GO terms associated with the mitochondria are enriched in the gene set comprising Cluster 6, which is also discontinuously upregulated, first at P7 and then from P21 to P28. We also found that Clusters 5 and 6 are enriched for GO terms associated with various metabolic processes and other homeostatic functions, suggesting that the middle of this time series represents the beginning of a shift from growth to homeostasis and a change in cell metabolism.

### Expression of “muscle” genes in the growing tendon

The detection of muscle-related transcription factors in our datasets may result from either the collection of fibroblast-fused muscle cells as *Scx*-lineage cells have been reported to fuse with myofibers and contribute to muscle [42], or from direct contamination from muscle in our samples. We show the specific expression pattern of each category of muscle-associated genes identified in Clusters 1, 2, and 5 (S3A Fig). Interestingly, there are subsets of muscle development genes that are differentially expressed in either direction throughout the time series. Both Clusters 1 and 2 show GO enrichments related to muscle development and differentiation (Fig 1D), but on closer examination this signal appears to be driven by different gene families. The muscle development signal in Cluster 2 appears to be largely driven by the expression of genes in the MEF2 family (*Mef2c*, *Mef2d*), while the Cluster 1 genes contributing strongly to this signal are myogenic regulatory factors (*Myf5*, *Myod1*, *Myog*). Because these genes are not widely studied in the tendon and were not expected to be expressed, we sought to replicate these results using alternative techniques for assessing gene expression. RT-qPCR on *Myf5* and *Myod1* using tendon RNA collected from a new cohort of *Scx-Cre;TdTom* mice, the same strain used to generate the RNA-seq libraries (see Methods; S3B and S3C Fig), replicated this result showing downregulation of both genes from early to late time points. Unlike Clusters 1 and 2, Cluster 5 is enriched for genes involved in regulating the assembly of contractile elements in striated muscle cells, specifically actin (e.g., *Acta1*, *Actn3*, *Tmod1*, *Tmod4*) and titin (e.g., *Tcap*). Although not enriched for muscle-specific processes, Cluster 4 contains many genes involved in the regulation of the actin cytoskeleton (e.g., *Acta2*).

### ATAC-seq identifies accessible chromatin regions in tendon cells

**Fig 2:**
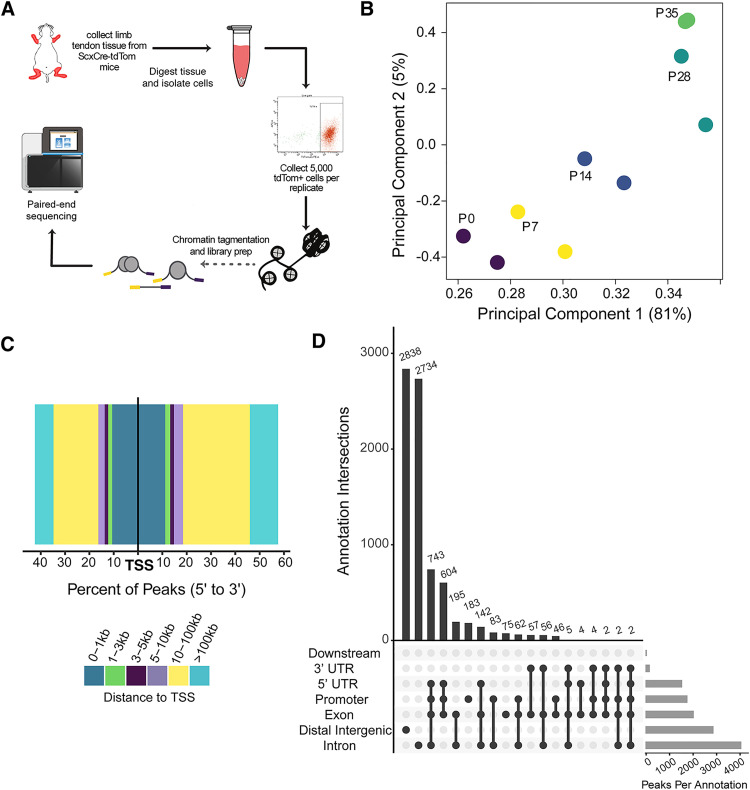
ATAC-seq of 5,000 sorted Scx-lineage cells detects differential chromatin accessibility during postnatal tendon development. **A)** Schematic of cell isolation and library preparation from mouse tendons. The genomesequencer9 icon from BioIcons, by DBCLS https://togotv.dbcls.jp/en/pics.html is licensed under CC-BY 4.0 Unported https://creativecommons.org/licenses/by/4.0/. **B)** PCA of normalized counts of ATAC-seq reads in peaks shows primary separation of timepoints along PC1. Early (P0 and P7), middle (P14), and late (P28 and P35) timepoints are further separated along PC 2. **C)** Bar plot showing distribution of consensus ATAC-seq peaks around TSS genome-wide. Peaks are binned and colored by distance from TSS. The percent of consensus peaks in each bin is plotted along the x axis in a 5’ to 3’ orientation, i.e., bars to the left indicate peaks upstream from a TSS and those to the right are downstream. **D)** Upset plot of the distribution and intersection of genomic annotations assigned to consensus ATAC-seq peaks showing that the majority of peaks are found in non-coding regions.

To map chromatin accessibility and putative TF-binding events throughout postnatal tendon development, we performed ATAC-seq on 5,000 FACS-sorted *Scx*-lineage (*Scx-Cre;TdTom*^+^) cells collected from tendons at the same six time points described above (Fig 2A). Due to insufficiencies in sequenced library quality, we had to discard both P21 replicates from the analysis. Because Tn5 transposase is known to preferentially cut certain sequences [43], we performed a control ATAC assay on naked, or genomic, DNA and sequenced this library along with those from each time point. We controlled for Tn5 sequence preference and other library preparation artifacts by filtering reads found in the control library from the data set for all downstream analyses (see Methods). All sequenced libraries are enriched for insert sizes < 250 bp, which indicates a large number of nucleosome free (<100 bp) and mono-nucleosomal (180–247 bp) regions [44]. Additionally, biological replicates for each time point are well correlated (Pearson correlation $\begin{array}{c} \;> \end{array}$ 0.8).

Localized regions of accessible chromatin (peaks) were identified for each replicate using the MACS2 peak calling algorithm [45] and a consensus peak set for all replicates was defined using the DiffBind package [46] in R yielding 67,438 consensus peaks that are accessible in both replicates of at least one time point. To determine differential accessibility of peaks over time, reads in peaks were counted for each biological replicate (see Methods) and the DESeq2 framework was implemented in DiffBind (see Methods). A PCA shows that the time points separate well along PC1, which explains 81% of the variance in the data set (Fig 2B). However, similar to the transcriptomic data discussed above, P28 and P35 fail to fully separate along PC1 suggesting minimal differences between these two time points. This is supported by the differential accessibility analysis, which finds only 8 differentially accessible (DA) peaks between P28 and P35. In contrast to the relative similarities between the two latest time points, 8,358 peaks were found to be DA at some point between P0 and P35 (adj. p < 0.05). The majority of these DA peaks are located distal to known transcription start sites (TSS) in non-coding regions of the genome: ~ 15% of DA peaks are found within the promoter of a gene and ~34% fall in a distal intergenic region, while less than 2% of DA consensus peaks are located within a known exon (Fig 2C and 2D). These results indicate that ATAC-seq identifies dynamic chromatin accessibility in non-coding regions of the mouse genome during postnatal tendon development.

### Integration of ATAC-seq and RNA-seq data

**Fig 3:**
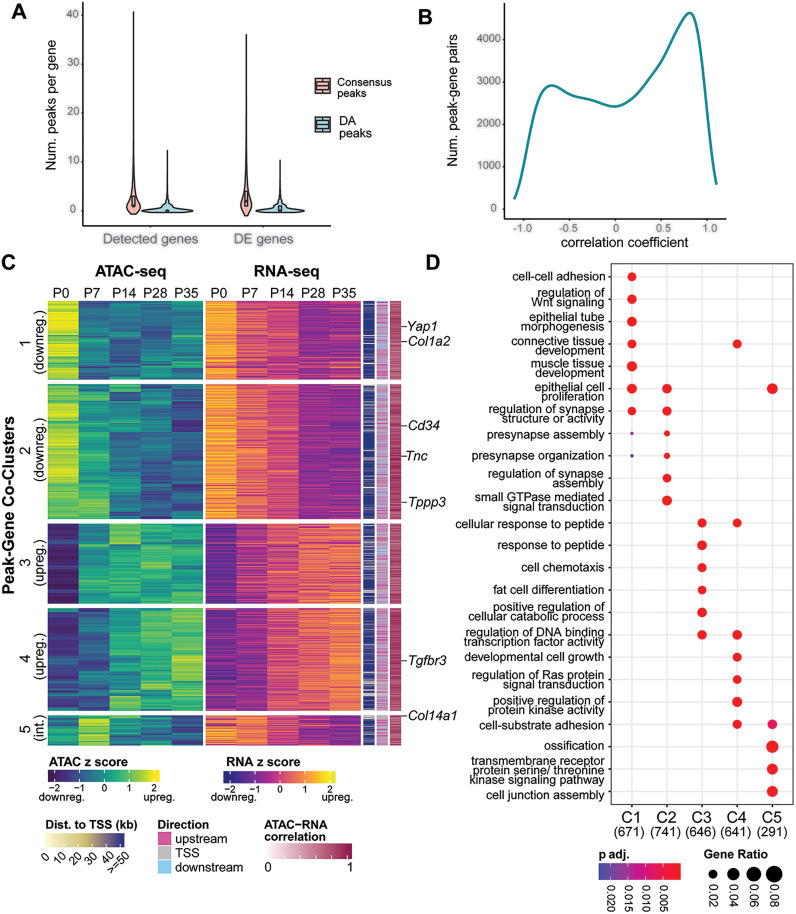
Integrative analysis of transcriptomics and chromatin accessibility reveals putative regulatory information about the postnatal transition in tendon cells. **A)** Violin plot showing the distribution of the number of ATAC-seq peaks assigned to a given gene. While most genes (either merely detected or DE) had ≥ 1 associated consensus peak, fewer genes had ≥ 1 associated DA peak. **B)** Density distribution of correlation coefficients calculated for gene expression and chromatin accessibility over time for all peak-gene pairs. **C)** Heatmap of ATAC-seq and RNA-seq peak-gene co-clusters determined using the PAM algorithm *with key developmental tendon genes annotated*. Z-scores were calculated from log2(x + 1)-transformed, quantile normalized CPM. Heatmap annotations are (from left to right): absolute distance of the peak from the nearest TSS; direction from TSS, i.e., upstream, downstream, or overlapping with the TSS annotation; correlation score (r2) for the peak-gene pair based on ATAC-seq and RNA-seq normalized counts across the time series. **D)** Dot plot depicting the top five enriched GO categories for each cluster. Bubble color represents the adjusted p value for the enrichment and bubble size represents the gene ratio for the GO term.

To test whether any of these DA regions could harbor functionally relevant *cis*-regulatory elements, we first examined the relationship between genome-wide chromatin accessibility and gene expression measured via RNA-seq across postnatal tendon development. ATAC-seq peaks were assigned to the nearest gene and Pearson correlation was computed between peak accessibility and gene expression for each peak-gene pair. Of the 6,193 DA peak-gene pairs for which gene expression was detected throughout the time series, 2,180 (~35%) were found to exhibit a positive correlation between accessibility and expression (Pearson correlation > 0.5) indicating potential transcriptional activation (enhancer) activity by these DA peaks (Fig 3B). Meanwhile, 1,335 (~21%) demonstrated a negative correlation (Pearson correlation < -0.5) (Fig 3B). Although we are interested in general transcriptional regulation throughout postnatal growth, the relationship between potential transcriptional activators and their targets is more straightforward. Because little is known about *cis*-regulatory control during tendon growth in general, we chose to focus on potential enhancer regions in downstream analyses. We applied the PAM algorithm [47] to the subset of positively correlated peak-gene pairs based on their accessibility and expression over time, which revealed three primary patterns of accessibility partitioned into five main clusters (Fig 3C). Similar to the transcriptomic clustering results, the majority of these peaks and their assigned genes show either monotonic increasing or decreasing accessibility and expression over time. Interestingly, this co-clustering analysis also found a group of peak-gene pairs that exhibit a specific, coordinated increase at P7 (Fig 3C), similar to the pattern reflected in Cluster 4 (expression downregulation over time) from the transcriptomic analysis (Fig 1D).

Next, we performed enrichment analyses on the integrated modules to determine potential patterns in gene function. Much like the transcriptomic results discussed above, the two modules that characterize a coordinated decrease in chromatin accessibility and gene expression over time (Modules 1 and 2) are both enriched for peaks associated with Wnt signaling related genes (*Wnt2*, *Fzd7*, *Lgr4*, *Wisp1*, *Wls*; p adj. < 0.005), as well as mesenchymal cell differentiation, proliferation, and organ growth (e.g., *Yap1*, *Igf1*, *Dlk1*, *Jag1*). Examination of known tendon ECM genes, *Col1a2* and *Tnc*, showed decreasing expression with associated genomic regions with less accessibility over time (S4 Fig). Interestingly, Module 1 is also enriched for peak-gene pairs associated with TGF-$\begin{array}{c} \beta \end{array}$ signaling (e.g., *Bmpr1a*, *Bmp2*, *Jun*; Fig 3), as is Cluster 5.

The two modules of integrated genomic data that represent upregulation over time (Modules 3 and 4) are both enriched for muscle-related GO terms, in keeping with the findings of the transcriptomic analyses, as well as MAPK signaling and cell migration (Fig 3). The earlier of these modules to become active, Module 3, is also enriched for peaks near genes involved in fat and immune cell differentiation, hematopoiesis, and the regulation of reactive oxygen species (e.g., *Runx1*, *Foxo1*, *Foxo3*, *Cebpb*), with considerable overlap between these GO terms. Module 4 is more specifically enriched for cell adhesion, integrin binding, and heart morphogenesis-related terms. Among the genes that appear to be driving this cardiac signal include *Sav1* and *Smad6*, which are known negative regulators of Hippo signaling (through Yap) and TGF-$\begin{array}{c} \beta \end{array}$ signaling, respectively. Module 5 is also enriched for peaks associated with genes involved in TGF-$\begin{array}{c} \beta \end{array}$ signaling.

### Motif analysis

Coordinated changes in accessibility and expression are suggestive of potential *cis*-regulatory activity at these non-coding loci, which is ultimately controlled by TF binding activity within these regions. Thus, we sought to identify which TFs may be capable of modulating the activity of these putative enhancers by searching for known DNA motifs within these peaks using the HOMER motif-finding algorithm [48]. Multiple motifs were identified in each peak, with the majority of motifs being found within 100 bp of the peak summit (S5A Fig) and slight variation in the distribution of the number of motifs found per peak across clusters (S5B Fig). Next, we performed enrichment analyses to identify motifs associated with specific accessibility and expression patterns in each co-cluster of DA peaks (q < 0.05). We found a number of motif enrichments that are shared between modules that change in the same direction with different timing (i.e., Clusters 1 and 2; Clusters 3 and 4), but very little overlap between peaks that become more accessible (Clusters 3 and 4) and those that become less accessible from P0 to P35 (Clusters 1, 2, and 5; S5C Fig).

**Fig 4:**
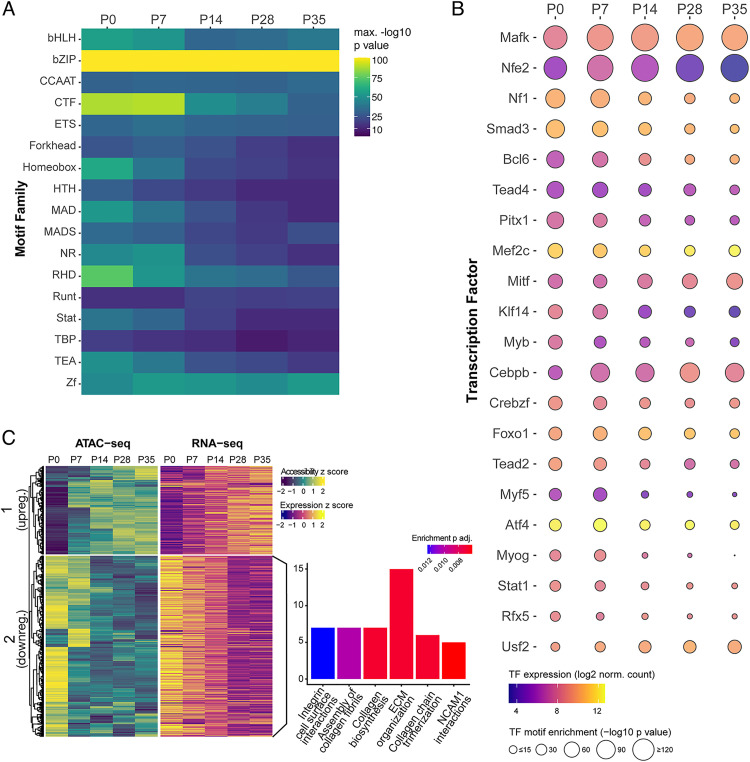
Motif analysis of differentially accessible chromatin regions identifies potential association of TEAD transcription factor with early postnatal ECM development. **A)** Heatmap of motif family enrichment within DA ATAC-seq peaks at each time point. **B)** TF motif enrichment within DA peaks and gene expression from P0 to P35. TFs were only included if they were expressed at minimum at one time point and with motif enrichment p < 10-15 for at least one time point. **C)** Clustered heatmaps of DA ATAC-seq peaks containing TEAD motifs and expression their nearest genes. Linked bar plot shows the top 6 pathway enrichments for cluster 2; no enriched pathways were identified for cluster 1. Only positively correlated peak-gene pairs were included in this analysis.

Binding motifs for TFs in the bHLH, CTCF, Homeobox, MAD, RHD, and TEA families are more enriched at earlier time points, showing an accessibility pattern consistent with Cluster 1 (Fig 4A). Specifically, motifs for Tead2, Tead4, and Smad3 show decreasing accessibility over time and, importantly, the genes encoding these TFs are also expressed at all examined time points (Figs 4B and S5C). The enrichment patterns of “muscle” transcription factor motifs mirrors what we found in our transcriptomic analyses: Myf5, Myog, and Myod motifs are enriched in peaks that are more accessible early (Figs 4B and S5C), while Mef2a motifs are enriched in peaks that become accessible later (S5C Fig). As our ATAC-seq analysis was performed on isolated *Scx-Cre;TdTom*+ cells, it is possible that these data originate from the *Scx*-lineage fibroblasts that fuse with muscle during development [42].

Because the primary model of enhancer function requires interactions between the proteins bound at both the enhancer and promoter [49], it is also important to know which TFs are capable of binding the putative target promoter of a given *cis*-regulatory region. To that end, we identified TF binding motifs in the annotated proximal promoter regions of the genes assigned to these putative enhancers (positively correlated DA peak-gene pairs) and tested whether similar TF motifs, and/or those with known interactions, were enriched in both (S5D Fig). Overall, we found fewer specific motifs enriched for each cluster (q < 0.05), but they largely reflected enrichment of the same protein families if not the same TFs (S5D Fig). Specifically, we identified enrichments of binding motifs for Tead2 (Cluster 4) and Tead4 (Clusters 1, 2, 4) in promoter-proximal regions of the putative target genes of these potential enhancers (S5D Fig). Based on the RNA-seq data from this study, *Tead2* is significantly downregulated from P0 to P35 and an RT-qPCR analysis on independent samples verified this finding (S6 Fig).

Because Tead2 and Tead4 binding motifs were identified as being enriched in both DA ATAC-seq peaks and their putative promoter regions in this data set, and both are expressed throughout the postnatal developmental time series, we narrowed our focus to the TEAD family of TFs. Subsetting to select only the DA peaks with TEAD family motifs and re-clustering the peak-gene pairs revealed two key patterns of accessibility and expression change from P0 to P35: upregulation (TEAD group 1) and downregulation (TEAD group 2) (Fig 4C). We performed a pathway enrichment analysis on each set of downstream genes and found that multiple ECM and collagen related pathways were enriched in TEAD group 2 (Fig 4C). However, no pathway enrichments were identified for TEAD group 1.

### Functional role of Yap signaling in postnatal tendon development

Given our findings that *Tead2* is downregulated in tendon tissue from P0 to P35 and Tead2 motifs are enriched in both ATAC-seq peaks that are DA at earlier timepoints and in the proximal promoters of their putative targets, we turned our attention to the broader context in which TEAD family TF binding could be functionally important during tendon postnatal development. Tead2 is a well-known co-factor of Yap/Taz; because Yap/Taz are unable to bind DNA directly, Tead co-factors play a vital role as effectors in Yap/Taz mediated Hippo signaling [50]. An RT-qPCR analysis of *Yap* and *Taz* expression showed that these central signaling factors are significantly downregulated at P28 compared to P0, and both genes are differentially expressed in the RNA-seq dataset as well (S6 Fig). Together with the results from our integrative sequencing analysis, this led us to hypothesize that Hippo signaling plays an important role in postnatal tendon development and growth, specifically with respect to tendon ECM.

**Fig 5:**
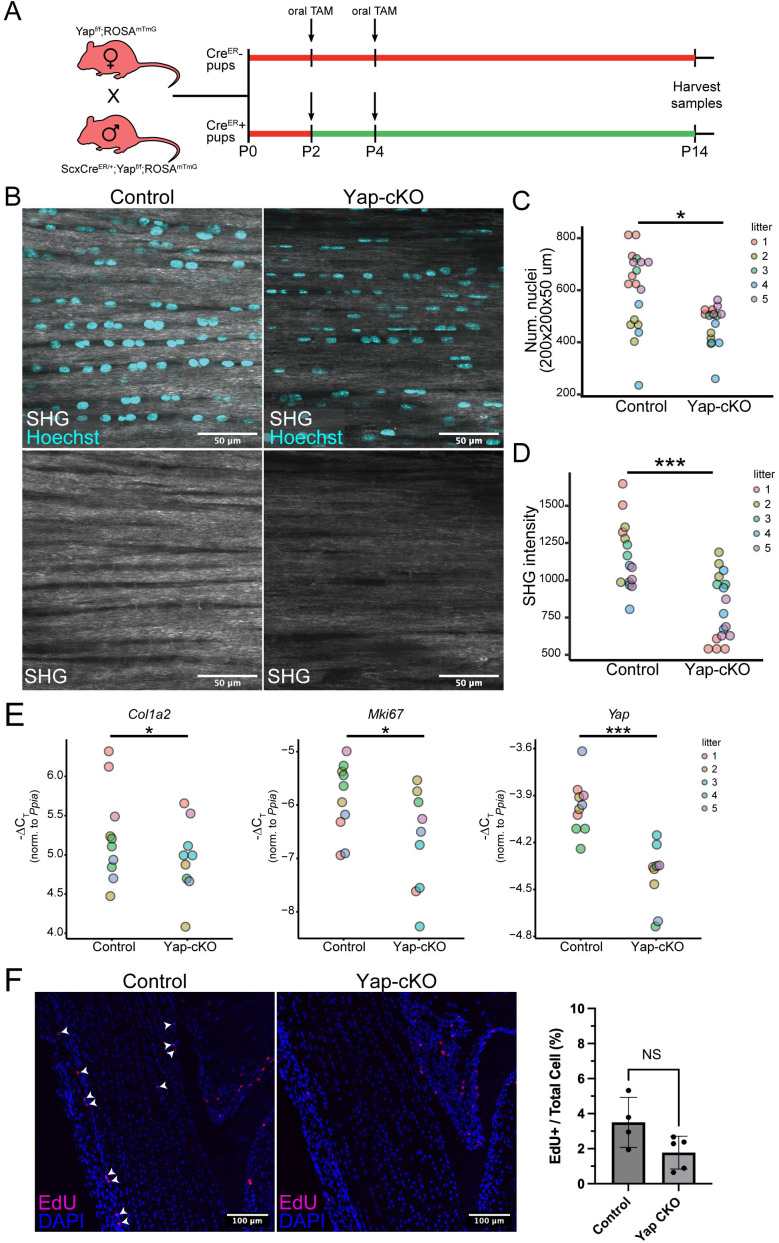
Neonatal deletion of Yap in Scx-lineage cells disrupts collagen production in the tendon during postnatal development. **A)** Schematic representation of mouse crosses to obtain Yap-cKO pups and wildtype littermates, as well as the tamoxifen dosing schedule. Mouse icon from PhyloPic, by Ben-Murrell undefined is licensed under CC0 https://creativecommons.org/publicdomain/zero/1.0/ **B)** Representative multiphoton images of control (Yap^f/f^) and Yap-cKO (Scx-CreER;Yap^f/f^) Achilles tendon in the sagittal plane at P14. Top panel shows the merged second harmonic (SHG) and Hoechst channels; bottom shows SHG signal alone. Laser parameters were kept constant for SHG signal acquisition for control and Yap-cKO samples within a litter. All litter mates were imaged in the same session. Scale bars are 50 µm. **C)** Yap-cKO Achilles tendons at P14 show a decrease in nuclear density (count per unit volume) compared to controls (p < 0.05). Quantification was performed on multiphoton z-stacks. **D)** SHG signal intensity is significantly lower in Yap-cKO Achilles tendons compared to controls (p < 0.001). Intensity was measured at P14 from multiphoton z-stacks. **E)** RT-qPCR shows decreased expression of Col1a2, Ki67, and Yap in and Yap-cKO tendon at P14 compared to controls. -∆CT represents expression of the target gene normalized to a reference gene (Ppia). * p < 0.05, ** p < 0.01, ***p < 0.001. **F)** Representative images of EdU staining in control (Yap^f/f^, left) and Yap-cKO (Scx-CreER;Yap^f/f^, middle) Achilles tendon at P14 (sagittal plane). Quantification of the percent of EdU+ cells in control (n = 4) and Yap-cKO (n = 5) Achilles tendons at P14 is shown to the right of immunofluorescence images (p = 0.0643).

To test this hypothesis, we conditionally deleted *Yap* in neonatal tendon cells using *Scx-CreERt2* (Fig 5A) and evaluated the tendon phenotype of *Yap*-conditional knockout (cKO) animals and littermate controls. We focused on Achilles tendons at P14 using multiphoton microscopy, which allowed us to assess ECM integrity via second harmonic generation (SHG) imaging. SHG signal intensity has been shown to reflect collagen structural maturity and density, thus making it a good proxy for high-collagen content ECM integrity [51]. We found that the SHG intensity of *Yap*-cKO tendons was significantly lower than that of littermate controls (Fig 5B and 5D; p < 0.001), indicating abnormal fibrillar collagen in the *Yap*-cKO tendons. Additionally, we found that nuclear density (number of nuclei per unit volume) was also significantly reduced in *Yap*-cKO tendons (Fig 5B and 5C; p < 0.05). To assess the effects of neonatal *Yap* conditional deletion on type I collagen production, we performed RT-qPCR on *Yap*-cKO and littermate control tendons and found that *Col1a2* transcripts were significantly depleted in the *Yap*-cKO tendons (Fig 5E; p < 0.05). This finding indicates that type I collagen production is hindered in the absence of Yap signaling, which could contribute to the observed change in SHG intensity. However, we cannot rule out the likely role of fibrillar collagen assembly and other important processes involved in the production of the tendon ECM. RT-qPCR also showed that expression of *Ki67*, a marker of cell proliferation, was significantly lower in the *Yap*-cKO samples compared to littermate controls (Fig 5E; p < 0.05), but analysis of EdU incorporation showed no significant difference in the percentage of EdU+ nuclei in *Yap*-cKO compared with control samples (Fig 5F). To determine if these changes in *Yap*-cKO samples at P14 affected the integrity and maturation of the tendon ECM, we performed mechanical testing and transmission electron microscopy on P28 Achilles tendon samples from *Yap*-cKO and control mice (S7 Fig). We observed no significant differences in the mechanical properties measured and the distribution of collagen fibril diameters in *Yap*-cKO compared with control samples, indicating that while loss of *Yap* in *Scx*-expressing cells affects tendon postnatal development at P14, these changes do not impact the later mechanical and ultrastructural properties of the tendon.

## Discussion

### Transcriptomics identifies differential expression of musculoskeletal genes

Using a transcriptomics approach, we identified genes differentially expressed during the postnatal transition from a proliferation-based growth program to one driven by ECM expansion. Most studies of postnatal tendons in mice have focused on changes to the ECM due to its important role in overall tendon tissue growth [20–22]. The ECM protein collagen makes up a large portion of the dry mass of tendon (65–80%), most of which is type I collagen [40]. Collagenous matrix production occurs in three phases: collagen molecule secretion and assembly into fibrils (fibrillogenesis); end-to-end fibril assembly to increase length; and lateral assembly to increase fibril diameter. We found that expression of the procollagen genes *Col1a1* and *Col1a2* peaks around P14 in accordance with our previous findings from RT-qPCR [23]. Expression of procollagen genes is indicative of collagen molecule production and secretion [52,53]. It has also been previously shown that *Col3a1*, *Lum*, and *Bgn* are involved in fibril assembly and growth and are downregulated with age [19,22,54], which is supported by our data as well (S2C Fig). In contrast to these downregulated genes, we found that *Dcn* is upregulated from P0 to P35 (p adj. < 0.01) and *Fmod* is upregulated from P0 to P21 (p adj. < 0.05), at which point expression appears to plateau (S2C Fig). These genes code for proteoglycans that bind fibrillar collagens and aid in regulation of lateral fibril growth [55–57], and the expression patterns shown here largely reflect those previously reported [19,22,23]

In contrast to the dynamic changes seen among ECM genes from P0 to P35, the embryonic tendon TF genes (*Scx*, *Mkx*, *Egr1*, *Egr2*) are not significantly DE at any point during this time series (p adj. > 0.05). This fact does not preclude their involvement in transcriptional activation during postnatal tendon development, it simply shows that they are not differentially regulated themselves. We previously showed via RT-qPCR that *Scx* is significantly upregulated at P14 relative to P0 and P35, and *Mkx* is significantly downregulated by P35 compared to P0, P7, and P14 [23]. This result differs from the RNA-seq results, however this discrepancy could be caused by multiple factors. First, RT-qPCR and RNA-seq measurements represent two different classes of data: RT-qPCR measures expression relative to some housekeeping gene(s) while RNA-seq measures absolute counts of transcript abundance and uses a model of transcriptome-wide expression variance to correct for biases in the data [39]. Second, the RT-qPCR assay was performed on a smaller sample size (n = 3 per time point) than the RNA-seq (n = 6–8 per time point), so it is possible that variation within this small sample was not representative of the true population variance. For this reason, it is notable that the RNA-seq *Scx* measurements have far higher within-group variability compared to other tendon genes (S2A Fig). High and low *Scx* expressing samples do not partition by sex, and none of the mice contain a *Scx* knock-out or knock-in allele in their genetic background. If such large variation in *Scx* expression is characteristic of postnatal tendon, the discrepancy between the RNA-seq results and those from the previous *Scx* RT-qPCR assay could be due to sampling bias from a highly variable population.

From the transcriptomics analyses, we were surprised by the number of differentially expressed genes that are typically associated with muscle. In fact, the size of these “muscle” gene cohorts in three expression modules (Clusters 1, 2, and 5) was substantial enough to influence the GO enrichment analyses (S3 Fig). Although RNA-seq Clusters 1 and 2 represent differential expression in opposite directions (downregulated vs upregulated, respectively), they are both enriched for muscle development and differentiation-related processes and functions. However, it seems that the direction of DE is partitioned by gene family. Genes in the Mef2 family of TFs (*Mef2c*, *Mef2d*) are upregulated from P0 to P35 (Cluster 2), while the myogenic regulatory factors *Myf5*, *Myod1*, and *Myog*, which are members of the bHLH TF family, are downregulated throughout postnatal development (Cluster 1). Meanwhile Cluster 5 is specifically enriched for muscle-related genes that are involved in contractile element regulation. *Myf5* and *Myod1* play a vital role in specifying the myogenic fate [58] and in regulating muscle stem cells during adulthood [59,60]. While *Myf5* gene expression in non-muscle tissues has been reported (e.g., brown preadipocytes, [61]), *Myod1* expression is restricted to the myogenic lineage. Therefore, a possible explanation for our findings is that our samples contained muscle cells, which could include those of the *Scx*-lineage [42]. *Mef2* genes are expressed in a variety of other tissue types [62]. *Mef2c* has been shown to promote smooth muscle cell proliferation [63] and can regulate the differentiation of cardiomyocytes [64], neurons [65], and hematopoietic cells [66]. Studies in Xenopus show *Mef2c* is co-expressed with *Scx* and cooperates with *Scx* to induce tendon genes [67]. Both Mef2c and Mef2d have roles in regulating cytoskeletal proteins [68]. Although the function of *Mef2c* and *Mef2d* transcripts in the developing tendon are unclear, it is possible that they originate from tendon cells and are involved in cytoskeletal organization of the maturing tendon cells.

### Shift in proliferative potential is correlated with transcriptome dynamics

Unsupervised clustering of significantly DE genes identified six gene co-expression modules that describe three broader patterns of expression. Cluster 1, which contains genes that are downregulated early (~P14-P21), is highly enriched for genes involved in cell cycle, and is consistent with previous findings that tendon cell proliferation decreases significantly from P0 to P21 [23]. Genes involved in Wnt (Clusters 1 and 3), Hippo (Cluster 1), and Hh signaling (Cluster 3) are also overrepresented in early time points. All three of these pathways can regulate cell proliferation and differentiation (reviewed in [69–71], and they have been previously investigated for their role in tendon progenitor cell specification and differentiation [72]. Exposure of early limb progenitor cells to Wnt maintains their proliferative abilities and increases the expression of soft connective tissue ECM genes such as *collagen 1* (*Col1a1*, *Col1a2*), *tenascin C* (*TnC*), and *Dcn* [73]. Others have also shown that Wnt signaling is sufficient to inhibit chondrogenic differentiation of mesenchymal cells within the developing limb [74]. More recent work has supported a role for Wnt signaling in tendon cell proliferation, gene expression, and adult tendon injury response [75,76]. Sonic hedgehog (Shh) along with FGF regulates axial tendon progenitor specification [77], and the expression of *Six1* in embryonic limb tendon [78]. Hippo signaling and Yap have been shown to transduce mechanical signals and alter transcriptional output to influence cell fate, proliferation, and organ size control [79–82]. Recent work has shown that Yap/Tax/Tead target genes and associated loci are differentially regulated upon the loss of mechanical tension [83]. Therefore, our results are consistent with gene signatures of cell proliferation that may be regulated by some combination of Wnt, Hh, and Hippo signaling.

### Changes in chromatin accessibility reflect transcriptomics and identify putative cis-regulatory regions

Overall, the modules of gene expression and accessibility changes are similar between the integrated differential chromatin accessibility and transcriptomic analyses alone. By analyzing the significantly differentially accessible (DA) regions in each module, we were able to identify putative transcription factors that could be binding and regulating these DA regions (Fig 4A and 4B). However, this analysis must be interpreted with caution as the identification of an accessible ATAC-seq region alone does not indicate that the region acts as an enhancer. Additional analysis and functional testing are necessary to confirm its role as a cis-regulatory element. For example, the presence of specific histone marks such as acetylated lysine 27 (H3K27ac) or monomethylation of lysine 4 (H3K4me1) on Histone H3 indicate an active enhancer whereas other chromatin marks (H3K9me3 or H3K27me3) would indicate inactive or silenced regions [84]. Operational testing includes transgenesis assays to determine if the region can drive specific spatiotemporal expression *in vivo* or CRISPR-mediated deletion of the region to test for effects on nearby gene regulation.

The integrative analysis demonstrates a correlation between decreasing Hippo and Wnt signaling and decreasing tendon cell proliferation over postnatal stages (Figs 1D and 1F, and 3C and 3D). Peaks that are more accessible early in postnatal development tend to be associated with Wnt signaling genes (Fig 1C) and contain AP-1 family binding motifs, including c-Jun and JunD, which are involved in the Wnt signaling pathway and cell growth (S5C and S5D Fig) [85–87]. The genes associated with these putative enhancers also contained a significant enrichment of AP-1 binding motifs in their promoter-proximal regions. Peaks near TGF-$\begin{array}{c} \beta \end{array}$ signaling factors were also found to be enriched in Modules 1 and 5, which represent early downregulation and P7-specific upregulation respectively (S5D Fig). Smad3 motifs are also significantly enriched in these peaks that become progressively less accessible over time (Modules 1, 2, and 5) (S5C Fig). Smad3 is one of two receptor-regulated Smad proteins and is a key transducer of canonical TGF-$\begin{array}{c} \beta \end{array}$ signaling [88]. Decreasing accessibility/expression of peak-gene pairs that are associated with TGF-$\begin{array}{c} \beta \end{array}$ and Yap1 signaling from P0 to P35 provides further evidence that these signaling pathways are specific to the proliferative period from P0 to P14, and that they may play a role in regulating this proliferation.

Our data may also point to some level of coordination between Wnt, TGF-$\begin{array}{c} \beta \end{array}$, and Hippo signaling in the transition from a highly proliferative to relatively quiescent tendon cell program and increasing matrix maturation. Crosstalk between the Wnt and TGF-$\begin{array}{c} \beta \end{array}$ signaling pathways has been demonstrated in multiple cell types. In vascular smooth muscle cells, TGF-$\begin{array}{c} \beta \end{array}$/Smad3 stimulates secretion of several Wnt ligands (Wnt2b, 4, 5a, and 9a), which promote proliferation by stabilizing *β*-catenin [89]. Other work in mesenchymal stem cells has shown that Tgfb1 stimulates expression of Wnt2, 4, 5a, 7a, and 10a [90,91] during chondrogenesis, and that Smad3 and Smad4 interact with *β*-catenin to activate the Wnt/*β* -catenin pathway in chondrogenesis [92] and osteogenic differentiation [93]. It has been shown in chondrocytes that TGF-*β* inhibits expression of *Axin1* and *Axin2* (negative regulators of Wnt) via Smad3, which promotes Wnt/$\begin{array}{c} \beta \end{array}$-catenin signaling [94]. Additionally, previous work has shown that Smad3 can regulate tendon ECM through physical interactions with Scx and Mkx [95], both of which are stably expressed during postnatal development (S2A Fig). Thus, it is possible that TGF-$\begin{array}{c} \beta \end{array}$ and Wnt signaling are regulating tendon cell proliferation and matrix formation.

As seen in the differential co-expression modules, DNA motifs for myogenic regulatory factors (e.g., Myod1) are significantly enriched within peaks that are more accessible early, while Mef2 motifs (e.g., Mef2a) are more prevalent in regions that become progressively more open during postnatal development (S4C Fig). While this evidence from DA chromatin does not definitively indicate binding of these myogenic TFs, it is suggestive that they may play a functional role in postnatal tendon development and maturation. As our ATAC-seq was performed on a sorted population of *Scx*-lineage cells, this should have minimized the possibility that these results are contamination driven (i.e., originating from non-tendon tissues). As *Scx*-lineage cells contribute to muscle and myotendinous cells, it is likely that our analysis includes these ‘hybrid’cell types [42].

We also identified DE genes that have been described to mark specific tendon cell populations in previous scRNA-seq datasets. For example, *Tppp3* and *CD34*, which are reported tendon progenitor cell markers [18,36], were found in Cluster 2 to be downregulated with associated genomic regions closing over postnatal time (Fig 3C). Other snRNA/ATAC-seq studies identified putative TF regulation of tendon progenitor cells by Klf family members, including Klf4 [30]. Our analysis identified Klf4 as having enriched motifs in distal and promoter proximal regions for cluster 4 genes, which are differentially increased over postnatal stages (S5C and S5D Fig). Analysis of *Tnc* and *Col1a2* identified putative enhancers that were differentially less accessible over the time series, which correlates to their decreasing gene expression (Figs 3C and S4). Therefore, our work provides a rich resource that can be used in conjunction with existing single cell and single nuclei sequencing datasets.

### Yap signaling in postnatal tendon development

Our analyses, using both RNA-seq and RT-qPCR, show that genes involved in Hippo signaling, specifically *Tead2*, *Yap*, and *Taz*, are decreased during the postnatal growth period (Figs 1D and 3C, and 4B, and S6). Furthermore, TEAD family motifs (especially Tead2 and Tead4 motifs) are enriched in both DA peaks that become less accessible from P0 to P35 (Fig 4A) and their putative target gene promoters (S5D Fig). We also found that genes associated with regions containing TEAD motifs are enriched for collagen ECM related pathways (Fig 4C). Lastly, we demonstrated that loss of Yap in neonatal *Scx*-expressing tendon cells affects both the ECM and nuclear density at P14, indicating an important role for Yap and Hippo signaling during early postnatal tendon development. However, analysis of Yap cKO tendons at later stages shows no effect on tendon mechanical properties and collagen fibril diameter. The resolution of these early phenotypic differences could be due to compensation by Taz, another transcriptional co-regulator of Hippo signaling, or the activation of other pathways and TF networks in tendon ECM maturation events. Supporting the former interpretation, recent work has shown that loss of both Yap and Taz affects tendon fibroblast gene expression with upregulation of genes involved in matrix degradation [83]. Although the Hippo pathway through Yap/Taz association with TEAD can regulate downstream transcriptional events, it can also interact and influence other well-known signaling pathways [96]. Yap and its cofactor Taz can interact with various Wnt transcriptional activators to regulate their cytoplasmic retention in a Wnt-dependent manner. In the context of fibrosis, Yap/Taz can form complexes with Smad2/3 and TEAD proteins, and the concentration of Taz influences nucleocytoplasmic shuttling of Smad proteins (reviewed in [97]. Thus Yap/Taz may be regulating gene expression through TEAD as well as other transcriptional regulators to control tendon gene expression and ECM maturation.

### Limitations

Although our work provides a new resource for understanding early postnatal tendon development, the study is not without its limitations. First, RNA-seq was performed on bulk tissue, and although tendon tissue was carefully microdissected, it is possible that other cell types were present. ATAC-seq was performed on sorted *Scx*-lineage cells, however, and shows relatively good concordance with the transcriptomics results, supporting the bulk RNA-seq results. The ATAC-seq was limited to samples of 5,000 cells per biological replicate. This constrains library diversity and increases the proportion of duplicate sequenced reads, thereby limiting our power to detect more minor changes in chromatin accessibility. Ideally, to achieve this level of sensitivity, each replicate would have required at least 50,000 cells, but the intrinsic hypocellular nature of tendon tissue makes this impossible without pooling many animals. We ultimately decided that, because pooling individuals into a “biological replicate” obscures true biological variation [98], it would be more appropriate to perform these assays on samples that originated from a single animal. However, this approach has its own caveats as pooled tendons from one individual contain different tendon types from both fore- and hindlimbs. Differences in developmental timing between fore- and hindlimbs are well documented [99]. Tendons also can differ in their developmental timing based on their anatomic location, tendon type (force-transmitting vs muscle anchoring; positional vs. energy storing), and even within the axis of the tendon itself, from its bone to muscle connection. Secondly, our biological sample size for the ATAC-seq assay is limited (n = 2 per time point). Tendon tissue is difficult to dissociate without harming the cells or changing their behavior. While a larger sample size (e.g., n = 4) would have been preferable, it was simply not feasible due to the high proportion of lost samples during the dissociation and/or sorting process caused by unhealthy cells. Previous studies have performed ATAC-seq on as few as 2 biological replicates and reported reproducible results [44,100], indicating that good quality data are possible from a limited sample number. To minimize spurious results, we constructed the consensus peak set using stringent criteria requiring a peak to be present in both biological replicates to be considered for differential accessibility; if a peak was only present in one replicate for a time point it was excluded from all downstream analyses. This likely inflates the number of false negatives in our results, but it is preferable to inflating false positives.

## Conclusions and Future Directions

Currently, we have a limited understanding of the gene regulatory networks and signaling pathways that control postnatal tendon growth and matrix maturation. As these stages also represent a turning point in regenerative to non-regenerative tendon healing and a significant transition in tendon mechanical properties, a molecular roadmap of the gene expression and genomic changes that occur during this period significantly advances our ability to test new pathways in these processes. Given our findings that Wnt, Hippo, and TGF-$\begin{array}{c} \beta \end{array}$ signaling appear to be downregulated in a coordinated fashion throughout postnatal development, it is possible that all three of these pathways play a role in the transition from a highly proliferative tendon cell program to a largely quiescent one. Our conditional deletion of *Yap* during the perinatal period resulted in modest, but significant, changes in tendon cell density and matrix gene expression with no later effects on tendon mechanical and ultrastructural properties, making it likely that its homolog, Taz, has similar functions and can compensate in its absence in regulating these processes. Targeting both Yap and Taz would further dissect the role of Hippo signaling in tendon development and maturation. Future functional studies targeting other components of this and other pathways will be vital for untangling the complicated network of interactions during tendon postnatal growth.

## Materials and methods

### Ethics statement

All mice used in this study were housed at the Center for Comparative Medicine at Massachusetts General Hospital (MGH) and experiments were approved by the MGH Institutional Animal Care and Use Committee (protocol #2013N000062).

*Scx-Cre* and *Scx-CreERT2* mice were provided by the Schweitzer lab [4,101]. *Gt(ROSA)26Sor*^*tm9(CAG-tdTomato)Hze*^ (Ai9) were purchased from the Jackson Laboratory (Jax 007909) and *Gt(ROSA)26Sor*^*tm4(ACTB-tdTomato,-EGFP)Luo*^ (referred to here as mTmG) were provided by the Rajagopal lab ([102]/Jax 007676). *Yap*^*flox*^ mice were provided by the Camargo lab [103]. To obtain *Scx*-lineage cells for ATAC-seq, male *Scx-Cre*^*+*^ mice were mated to female mice positive for the Ai9 Cre reporter allele to generate the *Scx-Cre;Rosa:TdTomato* mouse line (henceforth referred to as *Scx-Cre;TdTm*) to mark *Scx*-lineage cells.

For the *Yap* conditional knock-out (*Yap*-cKO) experiment, male *Scx-CreERT2* mice were mated to female mice homozygous for both the *Yap*^*flox*^ allele as well as mTmG. To perform conditional knock-out of *Yap*, all pups in each litter were given 10 μl of tamoxifen in corn oil (50 mg/ml) orally at P2 and P4. Pups were then genotyped at P10 for both *Scx-CreERT2* and floxed *Yap* alleles. An equal number of male and female mice were used for all experiments. Animals in this study were euthanized via decapitation (P0) or CO2 inhalation and subsequent cervical dislocation (P7 and older). In the *Yap*-cKO mouse model, Cre recombination in tendon cells was confirmed postmortem by visual inspection of mTmG signal in transverse tendon sections. Efficiency of floxed allele excision was evaluated by PCR using primers that amplify both wildtype and mutant (excised) regions in the *Yap* locus from tail tendon gDNA (see S1 Table for primer sequences). Finally, downregulation of *Yap* in cKO mice relative to wildtype littermates was confirmed via RT-qPCR using two sets of *Yap* primers that target different regions of the gene (see S1 Table for primer sequences). Only those mice that passed all three levels of *Yap*-cKO validation were used in subsequent analyses. Tamoxifen treated *Scx-CreERT2*– littermates served as wildtype controls.

### RNA Isolation

Extraction of intact total RNA from whole tendons was performed as previously described [23,104]. Briefly, distal hind limb and forelimb tendons were dissected from mice at weekly time points between P0 and P35 and submerged in cold TRIzol (Invitrogen 15596026) immediately following euthanasia. Multiple tendons from a single animal were collected in the same 1.5 mL tube containing 500 μl to 1mL of TRIzol and high impact zirconium 1.5 mm beads (Benchmark D1032-15). The volume of TRIzol used was dependent on the size of the sample. Tendons were first roughly chopped with clean microdissection scissors and then homogenized in two 180-second bouts of bead beating at 50 Hz in a BeadBug microtube homogenizer (Benchmark). Following homogenization, samples were stored at ${-\text{80}}^{\circ }$C until extraction using a combination of the TRIzol-chloroform method [105] and Zymo Direct-Zol system (Zymo Research R2050) with an on-column DNaseI digestion (Zymo Research E1010). RNA purity and quality were evaluated using spectrophotometry (NanoDrop 2000c, Thermo Scientific) and capillary electrophoresis (2100 Bioanalyzer and TapeStation 2200, Agilent), respectively. Concentration of each sample was measured via fluorometric quantitation (Qubit HS RNA assay, Invitrogen Q32852) and the final RNA product was stored at -${\text{80}}^{\circ }$C.

### RNA-seq library preparation and sequencing

RNA samples were excluded from RNA-seq experiments if RIN < 6.7 (threshold determined empirically), sample purity measures (260/280 and 260/230) were poor, and/or if the sample contained insufficient RNA for optimal library preparation. Between 7 and 9 biological replicates (i.e., tendon RNA from independent mice) per time point passed these quality measures, yielding a total of 46 samples spanning 6 time points. To minimize batch effects, all RNA-seq library preparation steps were completed in a single batch using the Apollo 324 NGS library prep system (IntegenX) and the PrepX mRNA library protocol (Takara Bio) in the Harvard Bauer Core Facility. First, mRNA was isolated from 1 μg total RNA by polyA selection (PrepX polyA 48, Takara Bio 640098) and checked for rRNA contamination using the mRNA 2100 Bioanalyzer chip and protocol (Agilent). The remaining mRNA for each sample was then reverse transcribed and purified using PrepX chemistry (PrepX mRNA 48, Takara Bio 640097). The resulting cDNA libraries were uniquely barcoded and amplified with 11 PCR cycles and then multiplexed into three pools of 16 samples for sequencing. Single end 75 bp reads were sequenced on an Illumina NextSeq 500 using a High-Output 75-cycle kit in the Harvard Bauer Core Facility. Sequenced libraries that achieved at least 20 million reads were used for downstream analyses.

### Tendon cell isolation and FACS sorting for ATAC-seq

Distal hind limb and forelimb tendons were dissected from *Scx-Cre;TdTm*^*+*^ mice at weekly time points from P0 to P35. Tendons were collected in a digestion buffer containing 0.2% collagenase type II (Worthington L5004176) in DMEM (Gibco 11965–092), coarsely chopped, and incubated in a shaking water bath at 37C. Midway through the incubation the digestion media was spiked with 0.2% collagenase type I (Gibco 17100–017) and 0.4% dispase (Gibco 17105–041). The digested tendons were then gently manually dissociated with a 20G needle and passed through a 30 $\begin{array}{c} \mu \end{array}$m pre-separation filter (MACS 130-041-407) to collect the cells. Tendon cells were washed with 10% horse serum in Hams F10 media (Gibco 11550–043) and finally filtered through the cell strainer cap of a FACS tube (Falcon 352235) for flow cytometry and cell sorting. Tendon cell suspensions were incubated with DAPI immediately before sorting for live/dead exclusion. TdTomato (TdTm) FACS gates were defined based on control TdTm^+^ and TdTm^-^ tendon cells that were collected and processed in parallel with the samples of interest. 5,000 TdTm^+^ cells were collected per replicate in 5% FBS/PBS. Control samples of 50 ng of naked DNA (i.e., DNA free of histones and DNA-binding proteins) from mice were also subjected to tagmentation and ATAC-seq library preparation in parallel with the tendon samples.

### ATAC-seq library preparation and sequencing

Cells were pelleted and washed in clean 1x PBS. Transposition was performed using the Fast-ATAC protocol [106] with the following changes: 2 μl of Tn5 transposase (TDE1 from Illumina FC-121–1030) was used in the transposition reaction and the reactions were incubated at ${\text{37}}^{\circ }$C in a shaking water bath for 35 minutes. Transposed DNA was purified using an Omega MicroElute Cleanup kit (Omega D6296) and eluted in 15 μl nuclease free water. The optimal number of PCR cycles for each library was determined via a qPCR side reaction [107]. The purified transposed fragments were then PCR amplified and barcoded as described in Buenrostro et al. [107], followed by bead purification (Omega M1386-01). Library quality was examined via capillary electrophoresis (2100 Bioanalyzer and TapeStation 2200, Agilent) and libraries were quantified using the KAPA library quantification system for qPCR (KAPA KK4824). ATAC libraries were multiplexed and paired-end 42 bp reads were sequenced on an Illumina NextSeq 500 using a High-Output 75-cycle kit in the Harvard Bauer Core Facility.

### RT-qPCR

Tendons were collected from *Scx-Cre;TdTm*^*+*^ mice (n = 4 per time point; 20 total) and RNA was extracted as described above. Because minimal differences were found between P28 and P35 in the transcriptomics analysis, we stopped collection at P28 for RT-qPCR validation. 1 μg of total RNA from each sample was reverse transcribed using the SSIV first strand synthesis system with oligo(dT)20 primers (Thermo Fisher 18091050). SYBR green (PowerUp SYBR, Applied Biosystems A25742) qPCR assays (S3 Data) were conducted in technical triplicate using 10 ng cDNA per reaction (final cDNA concentration = 0.8 ng/μl). Samples were amplified with target-specific primers (see S1 Table for primer sequences) using a LightCycler 480 II real time PCR system (Roche Diagnostics) as previously described [23].

### Multiphoton microscopy

Following euthanasia, mouse hind limbs were fixed in 4% PFA overnight at 4ºC. Fixed Achilles tendons were dissected from the limb, incubated in Hoechst 33258 (ThermoFisher Cat# H3569) nuclear stain (1:1000) for 2 hours at room temperature, rinsed in PBS, and mounted for whole mount multiphoton microscopy. Three Achilles samples were imaged per genotype (total n = 6) and images of at least different 3 regions of interest (ROI) within the tendon midsubstance were collected per sample. Laser parameters for each litter were calibrated on wildtype samples and used to image both control and *Yap*-cKO tendons to enable direct comparison of second harmonic intensity within a litter. Hoechst and second harmonic signal were acquired in 100 μm z stacks (0.5 μm step size) on an Olympus FluoView (FVMPE-RS) multiphoton laser scanning microscope using a 25X water immersion lens (XLPlan N 25X WMP) in the MGH Center for Regenerative Medicine Multiphoton Microscopy Core.

### Multiphoton image analysis

All multiphoton image analysis was conducted using FIJI software [108] with custom macros. Average second harmonic intensity was computed for each slice in the z stack, from which the maximum intensity was determined (S2 Data). Nuclei were counted (S1 Data) within a standardized volume (200 μm x 200 μm x 50 μm).

### *Yap* Conditional knockout phenotype characterization

Phenotype assessment of the *Yap*-cKO was conducted at postnatal day 28 (P28) on the Achilles tendon. Biomechanical testing (n = 8–15) was conducted following previously outlined testing methods [109]. In brief, the Achillies tendon was isolated from the murine hindlimb, preserving the calcaneus bone and gastrocnemius muscle. Calipers were used to measure the major and minor diameters of the Achilles at its thinnest location and cross-sectional area was estimated assuming an elliptical geometry. The sample was secured in custom grip fixtures, 3D printed using FormLabs Rigid10K. The calcaneal bone was placed within a recessed pocket, such that the Achilles’ natural orientation was preserved. The bone was potted with cyanoacrylate (Loctite 454) and a compression plate was added to fully secure the tissue. The muscle end was secured using compression clamps, with sinusoidally-shaped jaws to discourage tissue slipping. Samples were then mounted in a custom tensile device (10N load cell) for testing [110]. Tissue samples were pre-loaded to 0.01N to define tissue gauge length and then preconditioned for five cycles between 0.05 and 0.1N. Samples were held for 360 seconds and then ramped to failure (0.1%/s). From the load-deformation curve, stiffness was calculated by measuring the slope of the linear region using a linear least squares regression method that maximized the R^2^ value of the selected region. The maximum force was defined as the peak force recorded over the testing period. Samples were removed from downstream analysis if clear evidence of tissue slipping was observed. Mechanical properties are reported as mean ± standard deviation and differences were statically tested using a Welch’s t-test (S5 Data).

Transmission electron microscopy (TEM) was performed at the Shriners Hospital for Children in Portland, Oregon. Hindlimbs were fixed *in situ* and processed using standard methods [111]. A total of four *Yap-cKO* Achillies, and accompanying littermate controls, were analyzed. Cross-sections through the midsubstance of the Achilles tendon were examined and four non-overlapping representative areas were selected for imaging per sample (50,000x magnification). The minimum feret’s diameter were measured by hand to evaluate collagen fibril diameters and a total of ~1,300–1,500 fibrils were assessed per biological replicate. Fibril diameter distributions were statistically evaluated using a Kolmogorov-Smirnov test (S6 Data).

Changes in cellular proliferation following *Yap* CKO were assessed at postnatal 14 using EdU labeling. In brief, EdU (20mg/kg) was administered to P13 pups via subcutaneous injections and littermate CreER negative mice were used as controls. Animals were euthanized after 24 hours and limbs were fixed overnight in 4% paraformaldehyde. Limbs underwent serial sucrose infusions up to 30% sucrose before being embedded in OCT. Samples were cryosectioned at 12 µm and collected via established Cryotape protocols [112]. Click-iT EdU staining (Invitrogen C10340) was performed according to manufacturer’s instructions followed by a DAPI nuclear counterstain. Sections were imaged at the same approximate region of interest using confocal microscopy (40x mag.) using manufacturer recommended laser/filter settings. EdU+ cells were quantified relative to the total (DAPI+) cells within the tendon area. Three sections were evaluated and averaged for each biological replicate (n = 5–6). Statistical differences across experimental conditions were tested using an unpaired t-test (S4 Data).

### RNA-seq Data Processing and Differential Expression Analysis

Sequenced RNA-seq reads were demultiplexed, followed by quality filtering and trimming using TrimGalore [113]. After quality filtering, the final sample size was between 6 and 9 biological replicates per time point. A Salmon transcript index was built from the Ensembl mouse transcriptome (GRCm38v91) and transcripts were then quantified from quality-trimmed reads using Salmon [114] in mapping-based mode with the --seqBias and --gcBias flags enabled. When used, these options allow the Salmon algorithm to learn and correct for sequence specific and GC biases, respectively, in the data. Gene-level counts were then calculated with TxImport [115]. Genes with consistently low counts were excluded from the data set using automatic independent filtering implemented in DESeq2 [39,116]. Differential expression analyses were conducted using DESeq2 [39]. We defined two negative binomial generalized linear models of gene expression using RIN as a blocking factor:


$$log(\mu_{i,j})=\beta_{i}^{\text{0}}+(\beta_{i}^{RIN}x_{j}^{RIN})+(\beta_{i}^{TP}x_{j}^{TP})\;\;(\text{1})$$ (1)

$$log(\mu_{i,j})=\beta_{i}^{\text{0}}+(\beta_{i}^{RIN}x_{j}^{RIN})\;\;(\text{2})$$ (2)

where i=gene,j=sample. The full model (Equation (1)) includes both RIN and time point as predictor variables whereas RIN is the only predictor in the reduced model (Equation (2)). Using a likelihood ratio test (LRT) we compared the two models to identify significant genes that are explained by time point, but not RIN, in order to computationally account for any effects of RNA integrity on the results. The significance threshold for differential expression was set at p adj. < 0.05.

### ATAC-seq data processing and differential accessibility analysis

Demultiplexed paired end reads were trimmed using NGmerge [117] and aligned to the mouse genome using Bowtie2 [118]. Read pairs mapping to the mitochondrial genome were removed using removeChrom (https://github.com/jsh58/harvard) and PCR duplicates were filtered with the dedup tool in bamUtil [119]. Peaks were called with MACS2 in paired end BAM mode with the options -f BAMPE --nolambda --keep-dup all. Any peaks falling in the ENCODE defined blacklist for mm10 [120] and peaks called from the naked DNA control were deemed spurious and removed using bedtools [121]. Two successful biological replicates were obtained for all time points except P21, which was excluded from all analyses.

We defined a consensus peak set using the DiffBind R package [46] based on stringent requirements. In order for a peak to be included in the consensus set it had to be present in both biological replicates for at least one time point. Peaks that did not meet this criterion were deemed irreproducible and were excluded from all downstream analyses. We also constrained consensus peak widths to 500 bp. ATAC-seq peaks were annotated to genomic features in R using the ChIPseeker package [122] and were assigned to the nearest gene within a 100 kb window, defined as ± 50 kb from the consensus peak summit. DiffBind was used to count reads in these consensus peaks and compute peak differential accessibility (DA) using the underlying DESeq2 framework. The significance threshold was set at p adj. < 0.05.

### Clustering and functional enrichment

Prior to all clustering analyses, counts of significantly DE genes and DA peaks were normalized using the DESeq2 framework (median of ratios method) and scaled to library size to produce log2 counts per million (CPM). These counts were quantile-normalized and z-transformed. Pearson distance matrices were then calculated for the entire gene or peak set. Clusters were computed in R (R Core Team, 2019) based on this distance matrix and the normalized/transformed counts using the Partitioning Around Medoids (PAM) algorithm [47] implemented in the ClusterR package [123] with the fuzzy option enabled. Heatmaps of clusters were constructed using the ComplexHeatmap package [124] and genes (rows) within each PAM module were subclustered via average linkage hierarchical clustering to aid visualization. Functional enrichment analyses of peak and gene clusters were conducted in R using the clusterProfiler package [125] with significance thresholds set at p adj. < 0.01 and q < 0.05. The Benjamini Hochberg method was used for p value adjustment.

### Integrative analyses

In order to compare the patterns of gene expression and chromatin accessibility, DA peaks were clustered as described above (Methods, Clustering and Functional Enrichment) into five modules. Next, a matrix of normalized counts for each peak’s assigned gene was generated and this matrix was aligned with the matrix of normalized DA peak counts. We computed the Pearson correlation coefficient for each peak-gene pair using the ‘lineup’ R package [126]. For co-clustering of expression and accessibility patterns, the data set was filtered for a Pearson correlation coefficient > 0.5. This matrix of positively correlated of peak-gene pairs were re-clustered into five modules with the PAM algorithm. Gene lists for the final integrated clusters were used for functional analyses as described above (Methods, Clustering and Functional Enrichment). For each integrative cluster, known motifs were identified within peaks and putative target gene promoters using the HOMER motif finding algorithm [48]. Per cluster motif enrichment calculations based these on these motif predictions were facilitated by the ‘marge’ [127] and ‘valr’ [128] packages for R.

### RT-qPCR statistics

Cycle threshold ($C_{T}$) values for all targets were normalized to *Ppia* and the delta delta Ct method [129] was used to calculate relative gene expression for visualization. Relative expression is visualized as ${\text{2}}^{\Delta \Delta Ct}\pm$ standard deviation. Statistics were computed on the delta Ct values. Statistical differences among the time points were investigated using a Kruskal-Wallis rank sum test followed by a Dunn test with Benjamini-Hochberg correction to test for specific differences among pairs of time points. Statistics were performed on the delta Ct values (normalized to *Ppia*) for all target genes and samples (n = 4 biological replicates per time point). R statistical software (R Core Team, 2019) was used for all RT-qPCR data analysis, statistics, and visualization. Statistical analysis was performed using the implementation of the Kruskal-Wallace test in ‘stats’ (version 3.5.3) (R Core Team, 2019) and the Dunn test from ‘FSA’ (version 0.8.25; [130]).

### Multiphoton image analysis statistics

For both SHG intensity and nuclear density, differences between the controls and Yap-cKOs were assessed with linear mixed-effects models (LMMs) using the lmer function from lme4. The LMMs included both litter and sample as random effects.

### Software and computing environment

Processing and large-scale analysis of sequencing data was performed on the Harvard Odyssey computing cluster (centOS7). Python programs were run in Python version 2.7.12. R programs were run in R version 3.5.3 (R Core Team, 2019) and RStudio version 1.0.143 (RStudio Team, 2015). Data analysis and visualization in R was assisted by R packages included in the Tidyverse collection [131], ‘ggplot2’ [132], and ‘viridis’ [133].

## Supporting information

S1 Fig A-B) Principal Components Analysis (PCA) on normalized gene counts shows separation of samples by age along PC1 but not sample pool (A) or sex (B). C) Volcano plots showing differentially expressed genes between indicated timepoints.
(TIF)

S2 Fig A) Expression of known ‘tendon’ transcription factors, B) TGFβ and C) ECM genes measured with RNA-seq. Genes associated with tendon development are not significantly differentially expressed at postnatal stages (A). Analysis of TGFβ ligands and receptors shows that *Tgfb1* is differentially upregulated at P7 only, *Tgfbr1* is intermittently differentially expressed, and *Tgfbr3* is differentially upregulated from P0 to P35 (B). Expression of ECM related genes are significantly differentially expressed in unique directions during postnatal stages with *Dcn* and *Fmod* increasing gradually over time, *Col2a1*, *Col14a1*, *Col3a1*, and *Tnmd* decreasing over time, *Col1a1* and *Col1a2* peak in expression at P14, and *Bgn* is expressed at higher levels from P0-P14 after which its levels decrease (C). Kruskal-Wallis rank sum test followed by a Dunn test with Benjamini-Hochberg correction were used to test for specific differences among pairs of time points.
(TIF)

S3 Fig Expression of ‘muscle’ genes in the tendon during early postnatal development. A) Heatmap showing RNA expression of muscle-associated genes in Clusters 1, 2, and 5 with specific genes indicated. B) RT-qPCR for *Myf5* and *MyoD* show decreased expression over postnatal time, validating the results of the RNA-seq. Kruskal-Wallis rank sum test followed by a Dunn test with Benjamini-Hochberg correction were used to test for specific differences among pairs of time points.
(TIF)

S4 Fig (A,B) Example ATAC-seq coverage plots for two representative tendon genes, *Col1a2* (A) and *Tnc* (B). Normalized sequencing coverage for merged replicates are shown by timepoint aligned to gene annotations (mm10) and consensus peaks called from ATAC-seq read pileups. Red boxes highlight peaks that were called as differentially accessible and associated with each of these genes of interest.
(TIF)

S5 Fig A) Distribution of transcription factor binding motifs relative to differentially accessible ATAC-seq peak summits. B) Distribution of the number of motifs per peak across ATAC-seq centric clusters. C) Identification of motifs that are associated with specific ATAC-seq clusters demonstrates shared motifs among clusters changing in the same direction with developmental timing (Clusters 1 and 2 or Clusters 3 and 4) but little overlap in those changing in opposite directions (Clusters 3 and 4 compared with Clusters 1, 2, and 5). D) Enrichment of transcription factor binding motifs within the promoter regions of distal peak target genes.
(TIF)

S6 Fig A) RT-qPCR analysis of *Yap*, *Taz*, and *Tead2* shows a significant decrease in transcript levels over postnatal stages from P0 to P28. Statistical differences among the time points were investigated using a Kruskal-Wallis rank sum test followed by a Dunn test with Benjamini-Hochberg correction to test for specific differences among pairs of time points.
(TIF)

S7 Fig Structure-Function behavior following *Yap*-cKO. No significant differences were observed in **A)** animal weight, **B)** Achilles tendon cross sectional area, **C)** maximum force, or **D)** tissue stiffness between *Yap*-cKO (n = 6–9) and littermate CreER negative controls (n = 15–19) at postnatal day 28 (P28). Data represented as mean ± standard deviation. **E)** Representative transmission electron microscopy images of the tendon collagenous ultrastructure at P28. **F)** No significant differences were observed in the distribution of collagen fibril diameters following Yap-cKO (n = 4, both groups).
(TIF)

S1 Table List of RT-qPCR primers. (DOCX)

S1 Data This file has the nuclei counts for the control and Yap CKO samples shown in Fig 5C. (TXT)

S2 Data This file has the SHG measurements for the control and Yap CKO samples in Fig 5D. (TXT)

S3 Data This file contains the qPCR data for for the control and Yap CKO samples in Fig 5E. (XLSX)

S4 Data This file has the EdU counts for the control and Yap CKO samples shown in Fig 5F. (XLSX)

S5 Data This file has the mechanical testing data for the control and Yap CKO samples shown in S7A-D Fig. (XLSX)

S6 Data This file has the collagen fibril diameter measurements for the control and Yap CKO samples shown in S7 E-F Fig. (XLSX)
