# Supplementary figures and images for: Dynamic transcriptional and epigenetic changes define postnatal tendon growth
Source: PLoS Genet. 2025 Nov 18;21(11):e1011902. doi: 10.1371/journal.pgen.1011902 (PMC12626336; doi:10.1371/journal.pgen.1011902)

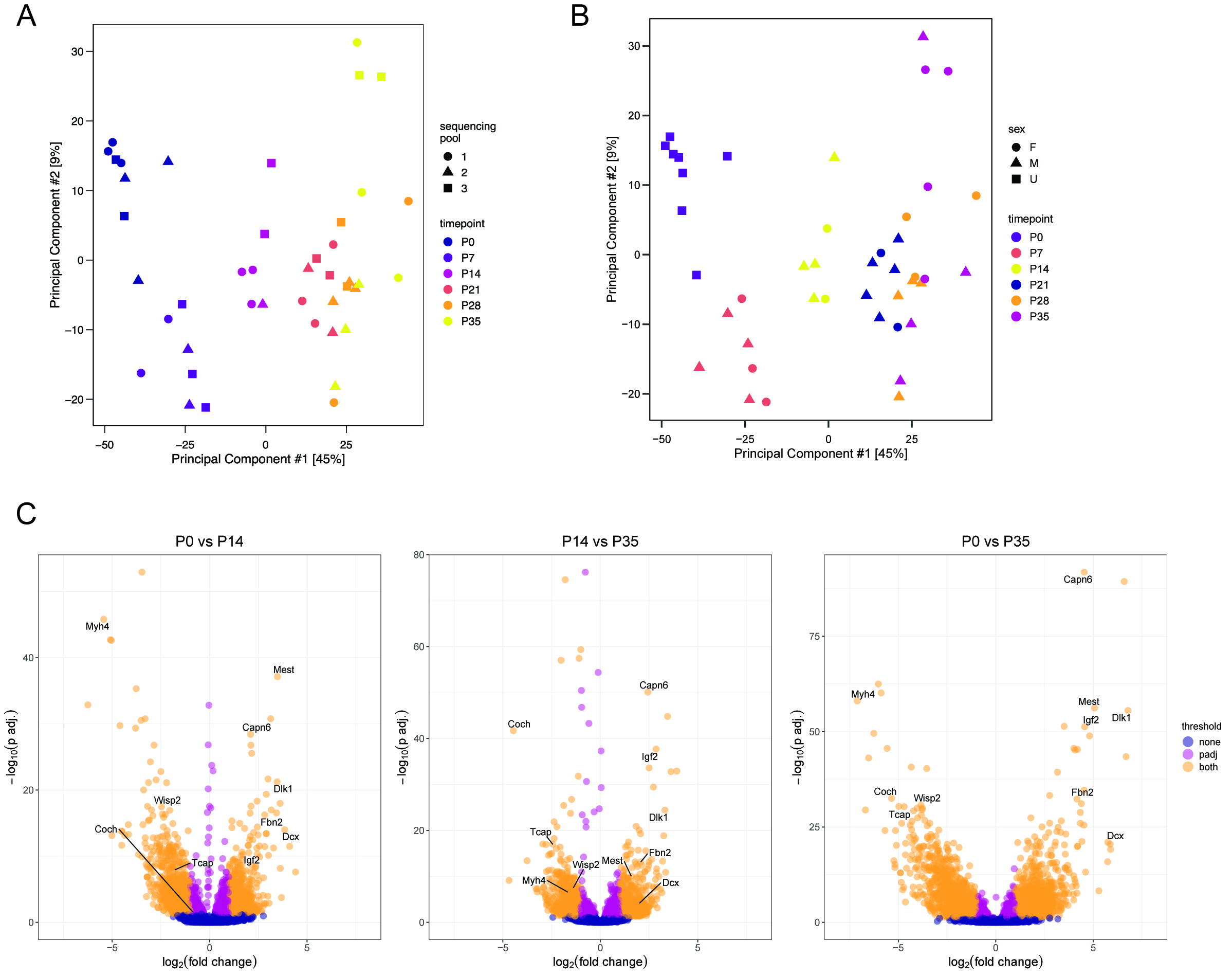

Supplement: S1 Fig — C) Volcano plots showing differentially expressed genes between indicated timepoints. (TIF) [file pgen.1011902.s001.tif]

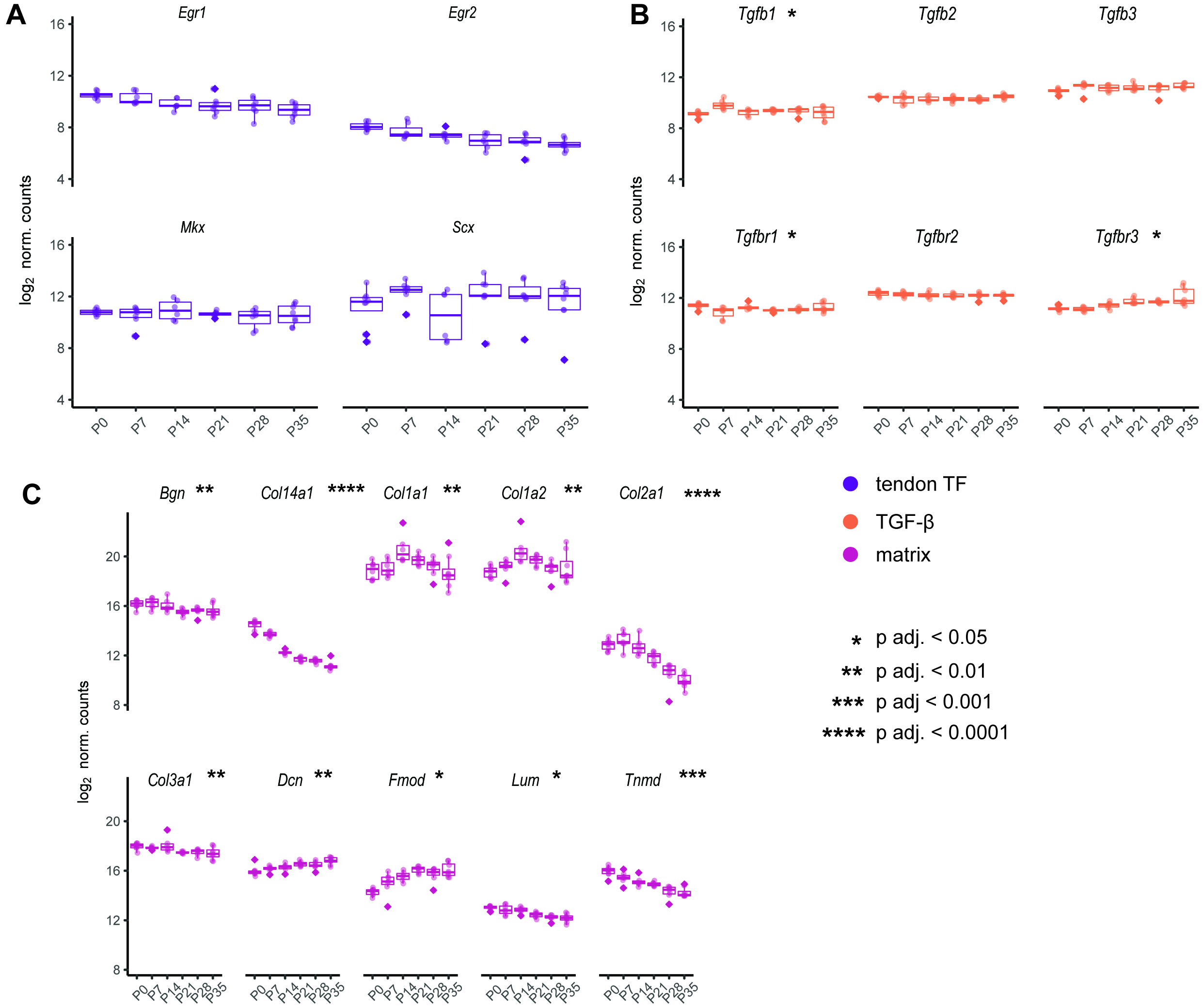

Supplement: S2 Fig — Genes associated with tendon development are not significantly differentially expressed at postnatal stages (A). Analysis of TGFβ ligands and receptors shows that Tgfb1 is differentially upregulated at P7 only, Tgfbr1 is intermittently differentially expressed, and Tgfbr3 is differentially upregulated from P0 to P35 (B). Expression of ECM related genes are significantly differentially expressed in unique directions during postnatal stages with Dcn and Fmod increasing gradually over time, Col2a1, Col14a1, Col3a1, and Tnmd decreasing over time, Col1a1 and Col1a2 peak in expression at P14, and Bgn is expressed at higher levels from P0-P14 after which its levels decrease (C). Kruskal-Wallis rank sum test followed by a Dunn test with Benjamini-Hochberg correction were used to test for specific differences among pairs of time points. (TIF) [file pgen.1011902.s002.tif]

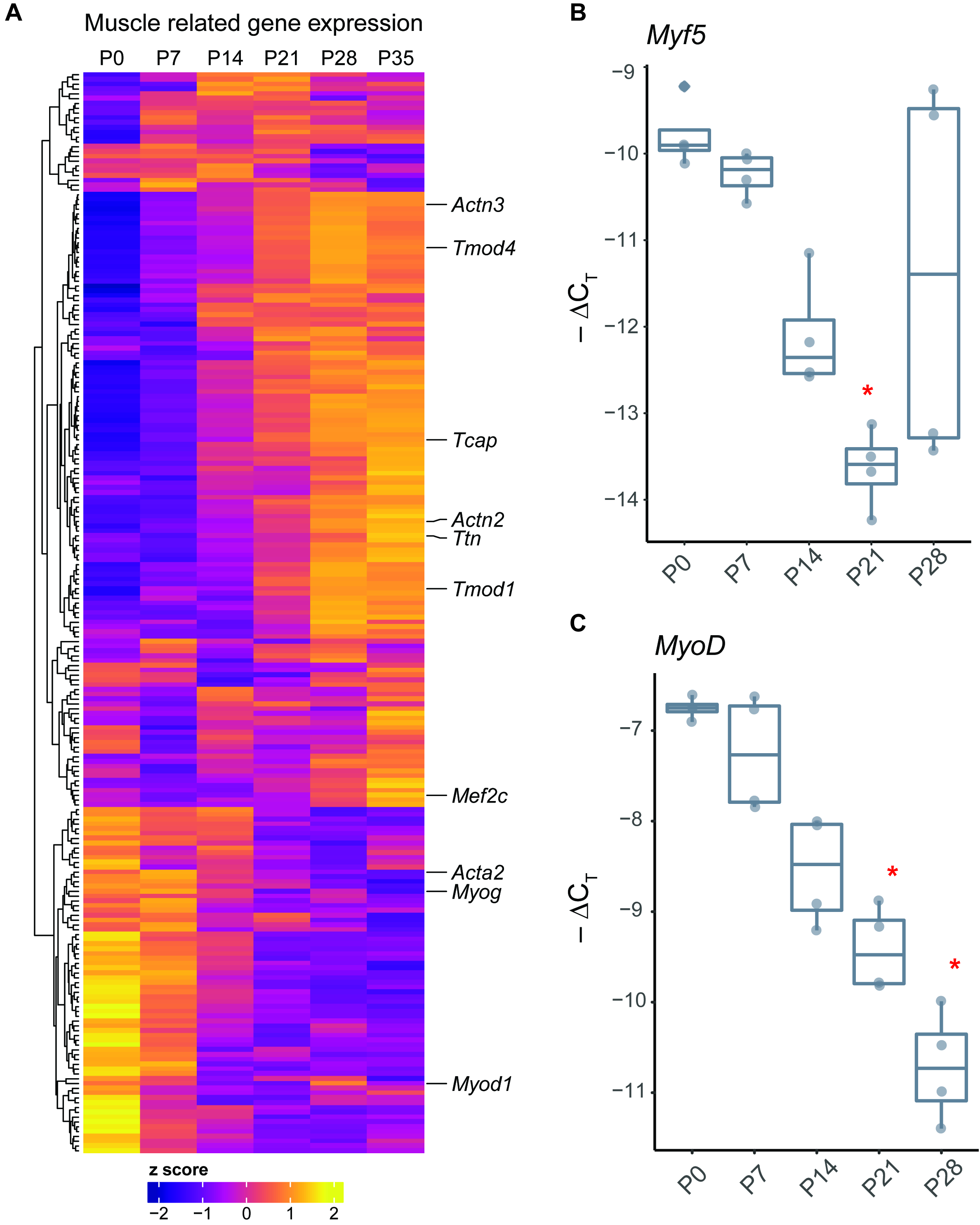

Supplement: S3 Fig — A) Heatmap showing RNA expression of muscle-associated genes in Clusters 1, 2, and 5 with specific genes indicated. B) RT-qPCR for Myf5 and MyoD show decreased expression over postnatal time, validating the results of the RNA-seq. Kruskal-Wallis rank sum test followed by a Dunn test with Benjamini-Hochberg correction were used to test for specific differences among pairs of time points. (TIF) [file pgen.1011902.s003.tif]

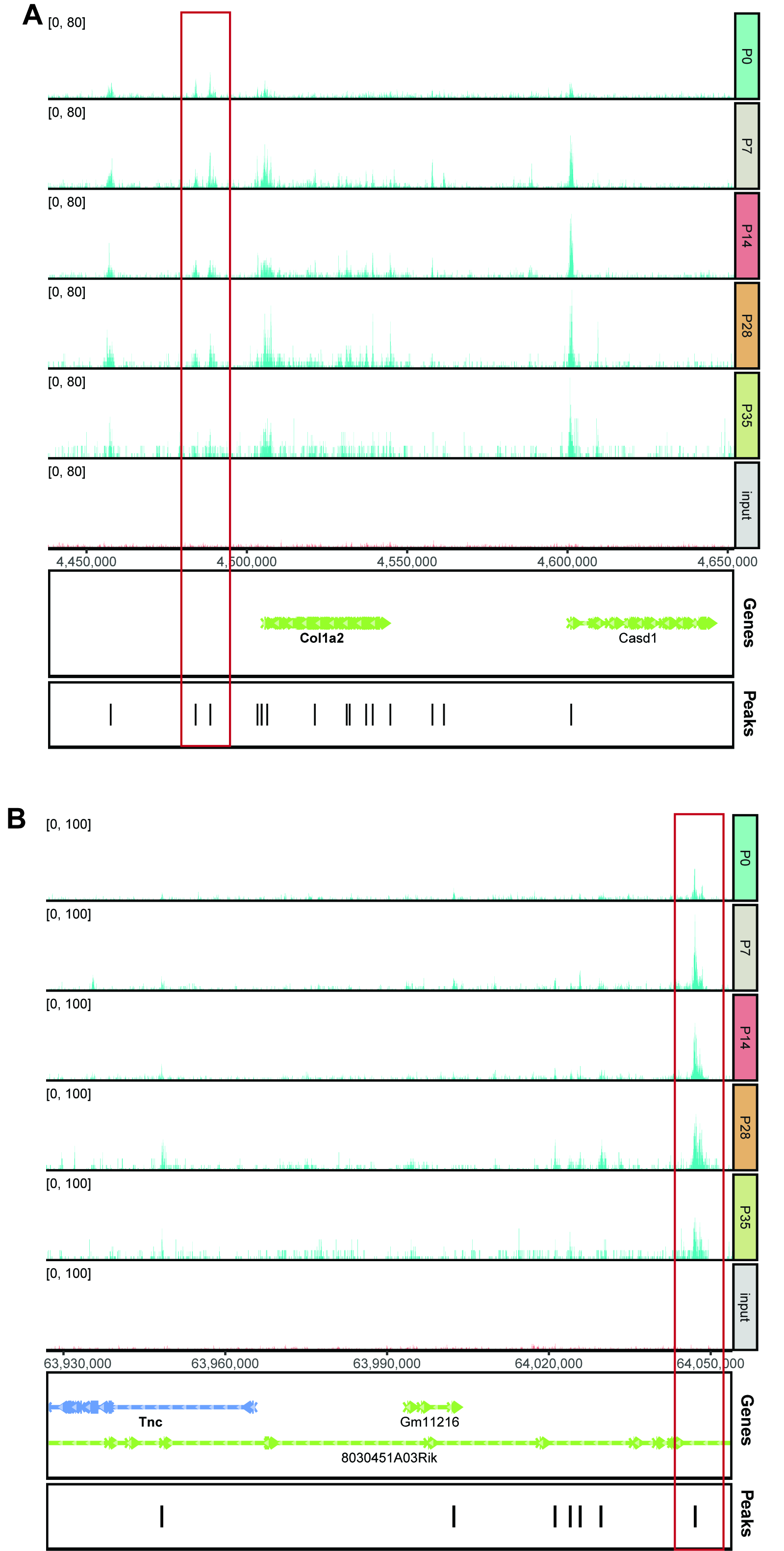

Supplement: S4 Fig — Normalized sequencing coverage for merged replicates are shown by timepoint aligned to gene annotations (mm10) and consensus peaks called from ATAC-seq read pileups. Red boxes highlight peaks that were called as differentially accessible and associated with each of these genes of interest. (TIF) [file pgen.1011902.s004.tif]

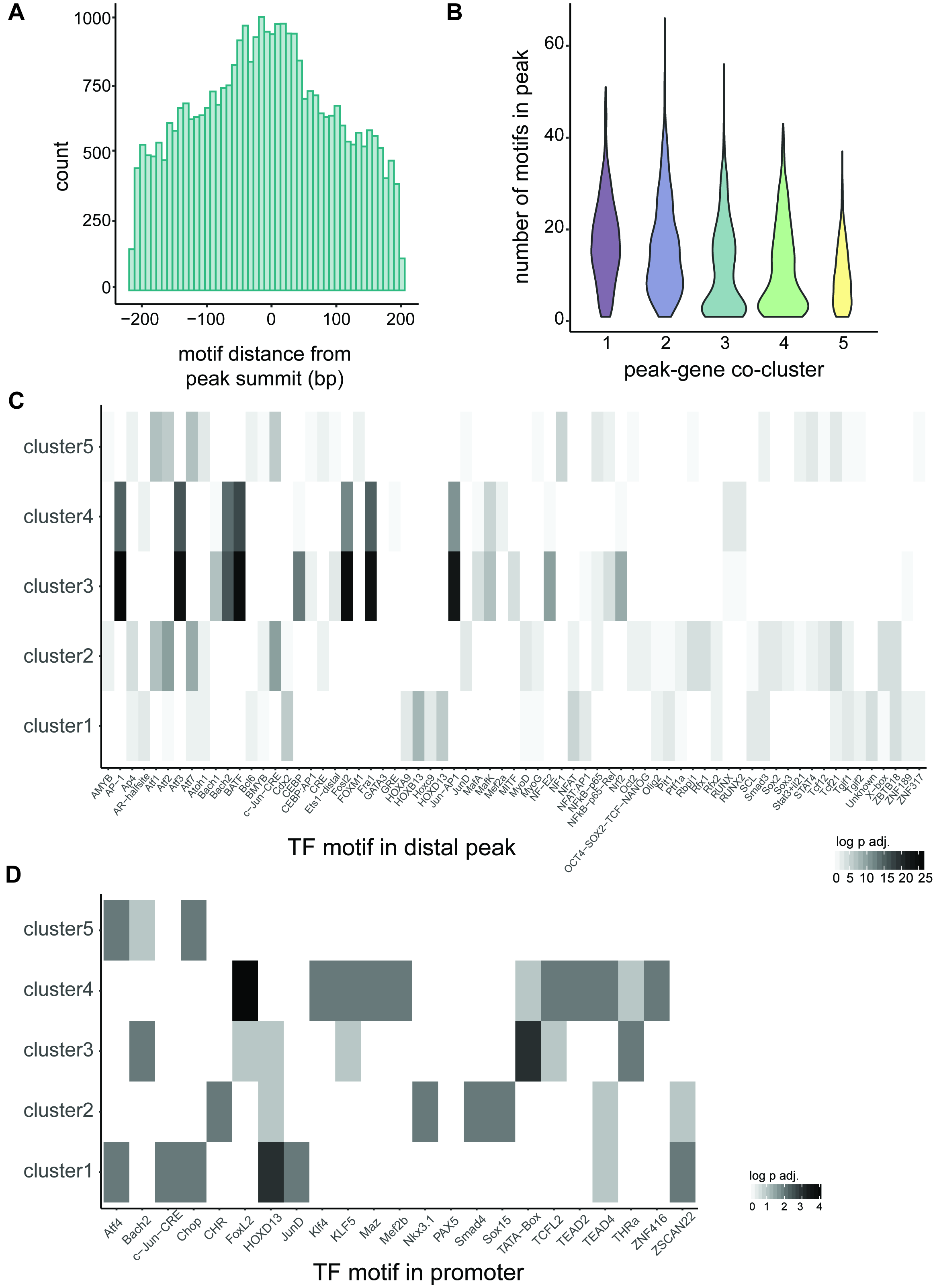

Supplement: S5 Fig — B) Distribution of the number of motifs per peak across ATAC-seq centric clusters. C) Identification of motifs that are associated with specific ATAC-seq clusters demonstrates shared motifs among clusters changing in the same direction with developmental timing (Clusters 1 and 2 or Clusters 3 and 4) but little overlap in those changing in opposite directions (Clusters 3 and 4 compared with Clusters 1, 2, and 5). D) Enrichment of transcription factor binding motifs within the promoter regions of distal peak target genes. (TIF) [file pgen.1011902.s005.tif]

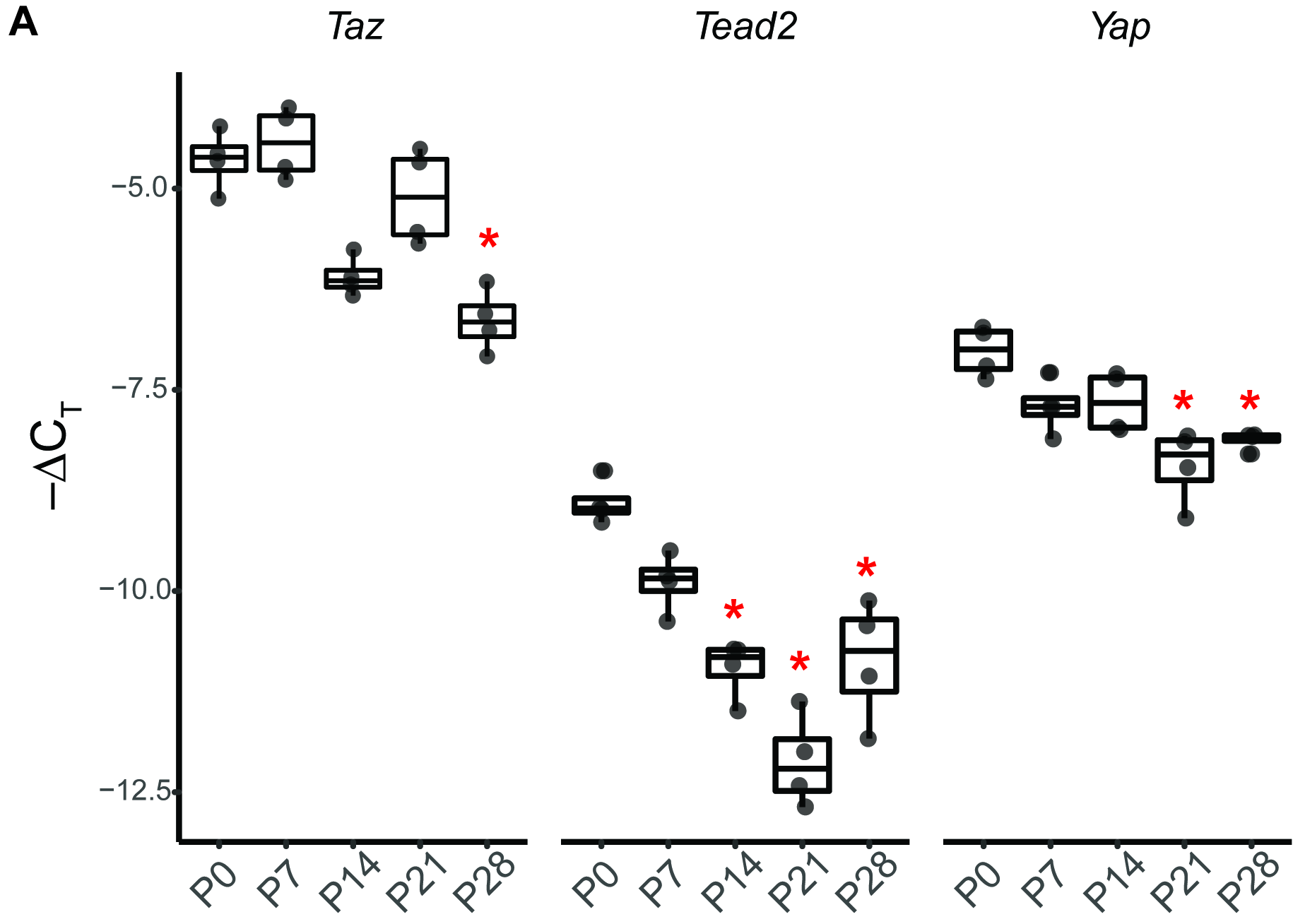

Supplement: S6 Fig — Statistical differences among the time points were investigated using a Kruskal-Wallis rank sum test followed by a Dunn test with Benjamini-Hochberg correction to test for specific differences among pairs of time points. (TIF) [file pgen.1011902.s006.tif]

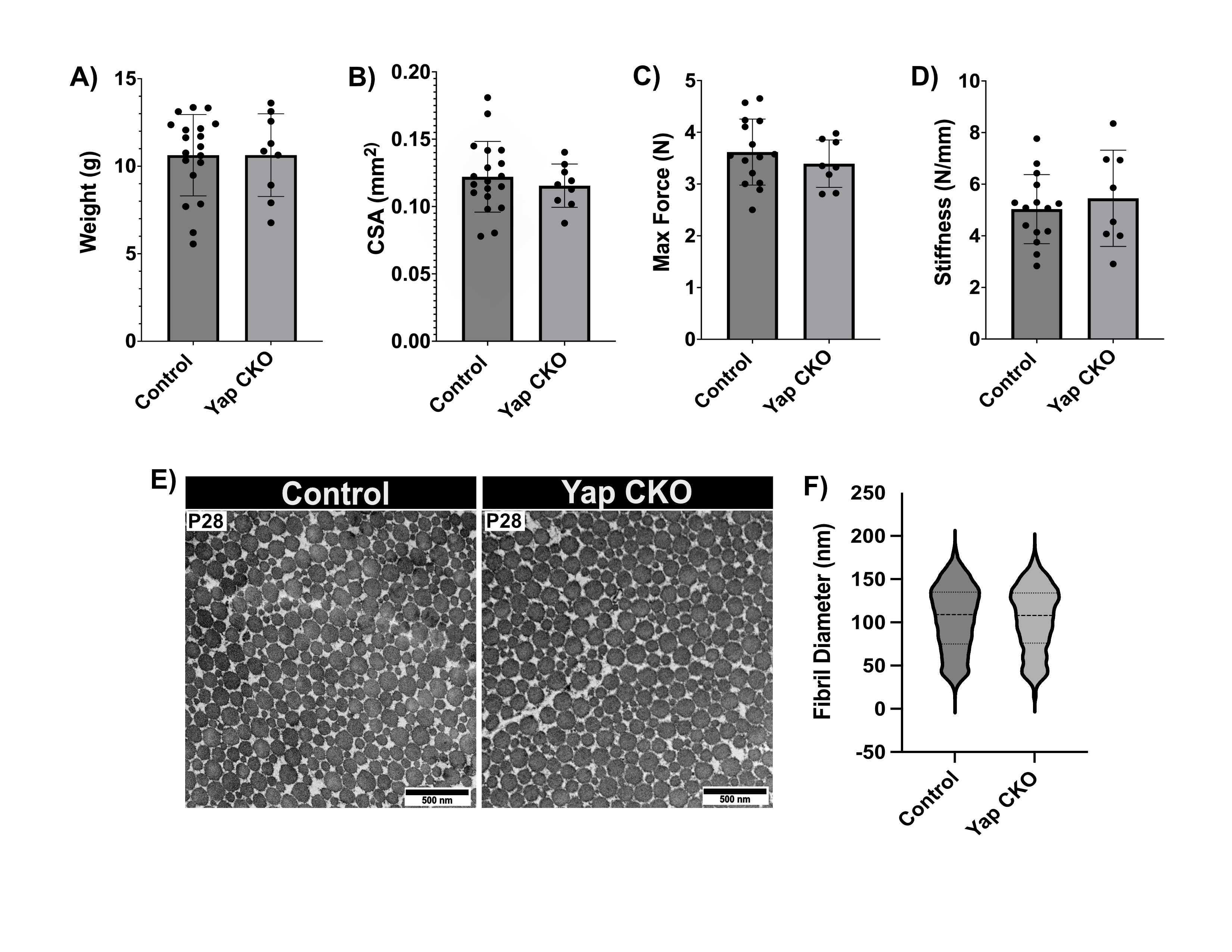

Supplement: S7 Fig — No significant differences were observed in A) animal weight, B) Achilles tendon cross sectional area, C) maximum force, or D) tissue stiffness between Yap-cKO (n = 6–9) and littermate CreER negative controls (n = 15–19) at postnatal day 28 (P28). Data represented as mean ± standard deviation. E) Representative transmission electron microscopy images of the tendon collagenous ultrastructure at P28. F) No significant differences were observed in the distribution of collagen fibril diameters following Yap-cKO (n = 4, both groups). (TIF) [file pgen.1011902.s007.tif]
