# Supplementary material for: Dynamic transcriptional and epigenetic changes define postnatal tendon growth
Source: PLoS Genet. 2025 Nov 18;21(11):e1011902. doi: 10.1371/journal.pgen.1011902 (PMC12626336; doi:10.1371/journal.pgen.1011902)
Supplement: S1 Table — (DOCX) [file pgen.1011902.s008.docx]

**Supplementary Table 1:** **List of RT-qPCR primers**

| Target Gene | Forward Sequence (5’–3’) | Reverse Sequence (5’–3’) | Primer Source |
| --- | --- | --- | --- |
| *Col1a2* | CCAGCGAAGAACTCATACAGC | GGACACCCCTTCTACGTTGT | (Mendias et al., 2008) |
| *Mki67* | AGCAAGCCAACAGAATTTCCAG | TATCTTGACCTTCCCCATCAGG | Self-designed using PrimerBlast |
| *Myf5* | CTGTCTGGTCCCGAAAGAAC | TGGAGAGAGGGAAGCTGTGT | (Shin et al., 2014) |
| *MyoD1* | TACAGTGGCGACTCAGATGC | GAGATGCGCTCCACTATGCT | (Hildyard and Wells, 2014) |
| *Ppia* | GGGTGGTGACTTTACACGCC | CTTGCCATCCAGCCATTCAG | Ruiz-Villalba et al. 2017 |
| *Taz* | GCCTGGCCTGCATTAAAATGG | CTTGCTTCAGAATTGGGCAGT | PrimerBank ID 21313658a1 |
| *Tead2* | GAGCCCCGACATTGAGCAG | CCGGCCATACATCTTGCCC | PrimerBank ID 7106433a1 |
| *Yap1* | AATGTGGACCTTGGCACACT | ACTCCACGTCCAAGATTTCG | (Szymaniak et al., 2015) |
| *Yap1* | CGCTCTTCAATGCCGTCATG | TGGAGAGGAGTGAGCTCGAA | Self-designed using PrimerBlast to target exons 1-2. |

**References:**

Hildyard, J.C., Wells, D.J., 2014. Identification and validation of quantitative PCR reference genes suitable for normalizing expression in normal and dystrophic cell culture models of myogenesis. PLoS Curr 6.

Mendias, C.L., Bakhurin, K.I., Faulkner, J.A., 2008. Tendons of myostatin-deficient mice are small, brittle, and hypocellular. Proc Natl Acad Sci U S A 105, 388-393.

Ruiz-Villalba, Adrián, et al. "Reference genes for gene expression studies in the mouse heart." Scientific reports 7.1 (2017): 24.

Shin, S., Suh, Y., Zerby, H.N., Lee, K., 2014. Membrane-bound delta-like 1 homolog (Dlk1) promotes while soluble Dlk1 inhibits myogenesis in C2C12 cells. FEBS Lett 588, 1100-1108.

Szymaniak, A.D., Mahoney, J.E., Cardoso, W.V., Varelas, X., 2015. Crumbs3-Mediated Polarity Directs Airway Epithelial Cell Fate through the Hippo Pathway Effector Yap. Dev Cell 34, 283-296.
